# Compatibility of Site-Specific Recombination Units between Mobile Genetic Elements

**DOI:** 10.1016/j.isci.2019.100805

**Published:** 2019-12-26

**Authors:** Shota Suzuki, Miki Yoshikawa, Daisuke Imamura, Kimihiro Abe, Patrick Eichenberger, Tsutomu Sato

**Affiliations:** 1 Research Center of Micro-Nano Technology, Hosei University, Koganei, Tokyo 184-0003, Japan; 2 Department of Frontier Bioscience, Hosei University, Koganei, Tokyo 184-8584, Japan; 3 Center for Genomics and Systems Biology, New York University, New York, NY 10003, USA

**Keywords:** Genetics, Molecular Genetics, Microbiology, Microbial Genetics

## Abstract

Site-specific recombination (SSR) systems are employed for transfer of mobile genetic elements (MGEs), such as lysogenic phages and integrative conjugative elements (ICEs). SSR between *attP*/*I* and *attB* sites is mediated by an integrase (Int) and a recombination directionality factor (RDF). The genome of *Bacillus subtilis* 168 contains SPβ, an active prophage, *skin*, a defective prophage, and ICE*Bs1*, an integrative conjugative element. Each of these MGEs harbors the classic SSR unit *attL-int-rdf-attR*. Here, we demonstrate that these SSR units are all compatible and can substitute for one another. Specifically, when SPβ is turned into a defective prophage by deletion of its SSR unit, introduction of the SSR unit of *skin* or ICE converts it back to an active prophage. We also identified closely related prophages with distinct SSR units that control developmentally regulated gene rearrangements of *kamA* (L-lysine 2,3-aminomutase). These results suggest that SSR units are interchangeable components of MGEs.

## Highlights

• Lysogenic phage-derived SSR unit is sufficient to drive SSR of ICE and vice versa
• Defective prophage-derived SSR unit can drive the excision of the active lysogenic phage
• Closely related prophages with distinct SSR units control each gene rearrangements
• Correspondence between MGEs and their cognate SSR units is not absolute

## Graphical Abstract

**Figure:**
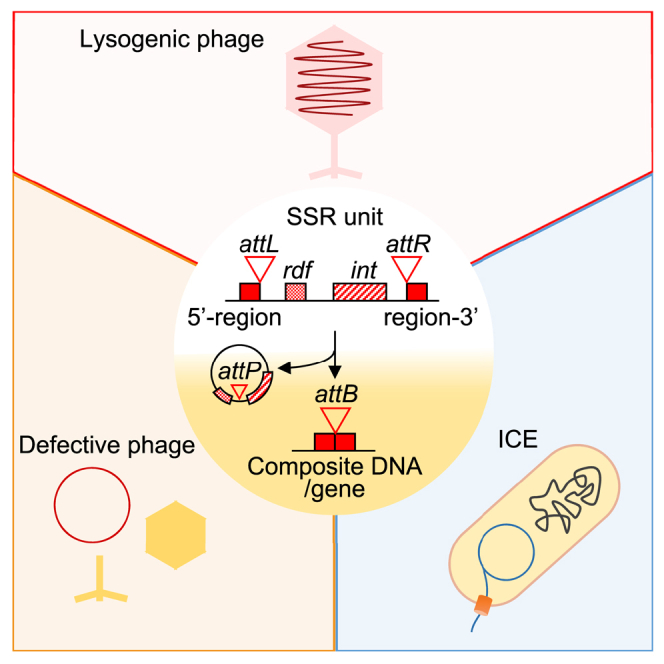

## Introduction

Bacterial viruses (bacteriophages or phages) infect bacterial host cells by injecting their genetic material. Phage virions consist of a protein coat that protects a DNA or RNA genome. Lytic (virulent) phages cause host cells to lyse after production of viral particles. Lysogenic (temperate) phages can switch between the dormant state (prophage), where the phage genome is integrated into the host chromosome, and the productive state, following excision of the phage genome from the chromosome. The loci where lysogenic phages integrate into the host genome are known as attachment (*att*) sites. For example, the bacteriophage λ genome, which carries an *attP* site, integrates in the genome of its host *Escherichia coli* at the *attB* site located between the *bio* and *gal* operons (Campbell, 1962, Landy and Ross, 1977). Similarly, SPβ from *Bacillus subtilis* has an attachment site within *spsM*, a gene required for spore polysaccharide synthesis (Abe et al., 2014, Abe et al., 2017b).

The basic genetic unit of site-specific recombination (SSR) systems in the genome of an integrated mobile genetic element (MGE) is *attL*-*int*-*rdf*-*attR* (SSR unit) (Groth and Calos, 2004). The phage-bacteria junctions, *attL* (left) and *attR* (right), are hybrids of the *attP* and *attB* sites. Attachment sites are recognized by an integrase, which either catalyzes phage integration (by recombination of *attP* and *attB*) or, conversely, excision of the DNA comprised between *attL* and *attR*. In addition, excision reactions require phage-encoded small proteins, known as recombination directionality factors (RDFs) (Ghosh et al., 2006, Fogg et al., 2014, Merrick et al., 2018). Integrases can be categorized into Ser (Large Ser-type Recombinase; LSR)- or Tyr-type families. Each type, however, uses common features to promote recombination between specific *attB* and *attP* sites. The bacteriophage λ integrase, Int, belongs to the Tyr-type family, whereas SprA (the SPβ integrase) belongs to the LSR-type family. Each excision reaction requires an RDF, known as Xis for λ and SprB for SPβ (Groth and Calos, 2004, Laxmikanthan et al., 2016, Abe et al., 2014, Abe et al., 2017b, Olorunniji et al., 2017). Integrative conjugative elements (ICEs) are another type of bacterial MGEs that can be transferred between cells by conjugation and subsequent integration via the SSR mechanism (Johnson and Grossman, 2015). In the *B. subtilis* ICE, ICE*Bs1*, integration and excision reactions are catalyzed by a Tyr-type integrase (Int_ICE*Bs1*_) and an RDF (Xis_ICE*Bs1*_), respectively (Lee et al., 2007).

Yet, in bacterial genomes like *B. subtilis* strain 168, these SSR units can be carried simultaneously by active prophages, ICEs, defective prophages, or non-prophage-like elements. Inactive prophages are often observed in genomes of spore-forming bacteria. Many of these are found in sporulation-specific genes (Stragier et al., 1989, Sato et al., 1990, Serrano et al., 2016). A classic example is the inactive prophage *skin*, which interrupts *sigK*, a gene encoding a mother cell-specific σ factor. The mother cell is one of the two cell types (the other being the forespore) generated after asymmetric division of the sporulating cell. Importantly, both cell types receive identical copies of the bacterial genome after division, but because the mother cell eventually lyses and the forespore matures into a spore, the spore genome can be viewed as the germ cell genome that will retain the inactive prophage. Several other genes specifically expressed in the mother cell of spore forming bacteria are known to be interrupted by MGEs. In *B. weihenstephanensis*, these include *spoVFB* (dipicolinate synthase subunit B gene) and *spoVR* (involved in spore cortex formation) (Abe et al., 2013). Similarly, *gerE* (encoding a mother cell-specific transcription factor) is interrupted in *B. cereus* (Abe et al., 2017a).

Spores of *Bacillus* species are usually produced in response to nutrient deprivation. Their dormant state is sustained until environmental conditions become favorable again for growth. Sporulation is an elaborate developmental process with well-defined temporal stages in the differentiation of the mother cell and forespore. Each cell engages in specific gene expression programs governed by a cascade of cell-specific sigma factors (σ^F^ and σ^G^ in the forespore and σ^E^ and σ^K^ in the mother cell) that control the expression of sporulation genes temporally and spatially (Bassler and Losick, 2006, Bate et al., 2014, McKenney et al., 2013, Shapiro and Losick, 2000). The sigma factor σ^K^ is the last sigma factor to be expressed in the sporulation cascade. As mentioned above, it is encoded by the composite gene *sigK* (5′-*sigK* and *sigK*-3′) interrupted by the defective prophage *skin*. To reconstitute a functional *sigK*, excision of *skin* is necessary (Stragier et al., 1989, Takemaru et al., 1995). This defective prophage contains its own SSR unit recognized by an Int_*skin*_ (SpoIVCA), which promotes excision (Sato et al., 1990, Kunkel et al., 1991). This process also requires an RDF (Skr, this work), whose expression levels are controlled by the mother cell-specific sigma factor σ^E^ (Sato et al., 1994). Mutations in *spoIVCA* cause sporulation defects because *sigK* remains interrupted by *skin* and the σ^K^-dependent genes fail to be expressed. Since the excision event is limited to the mother cell genome, *skin* is transferred vertically to the progeny through the spore (Sato et al., 1990). Similarly, many mother cell-specific genes in various strains and species (*sigK*, *spoIVFB*, *spoVR*, *spsM*, and *gerE*) are split by an element that carry their cognate SSR unit. Although some *rdf* genes have not yet been identified, excision processes are likely mediated by an individual integrase and its cognate RDF. It was recently discovered that the sporulation gene *spsM* was interrupted by an active prophage, SPβ, carrying the SSR unit *attL*-*sprB* (*rdf*)-*sprA* (*int*)-*attR*. The timing of phage excision is controlled by *sprB*, whose expression is dependent on stress-inducible and mother cell-specific promoters (Abe et al., 2014). Thus, the SSR unit responds to two pathways (i.e., stress and sporulation) that can trigger SPβ excision. Promotion of gene reconstitution by SSR units is, however, not limited to sporulation genes. For instance, these units have been described in MGEs that interrupt nitrogen fixation genes (*nifD*, *fdxN*, and *hupL*) (Golden et al., 1985, Golden et al., 1987, Carrasco et al., 1995, Carrasco et al., 2005). There are interesting parallels with the SSR units that are active during sporulation, considering that these elements were also excised in a developmental process, and heterocyst formation in the cyanobacterium *Anabaena* sp., where reconstitution of nitrogen fixation genes is similarly limited to one terminal cell type. Recently, Rabinovich et al. (2012) showed that the *comK* gene of *Listeria monocytogenes* was also interrupted by a prophage and that the prophage was excised during invasion into mammalian cells, thus allowing the bacteria to escape from phagosomes and colonize the host cell cytoplasm. With these examples in mind, we hypothesize that, because SSR units operate via a common recombination mechanism, they are independent of the MGE they reside in and could promote recombination in a variety of related elements.

Accordingly, SSR units could be transferred as independent units between lysogenic phages and ICEs. Here, we demonstrate that *B. subtilis* SSR units are compatible among lysogenic phages and ICEs. Our experiments also show that SSR units from a defective prophage and from an ICE can rescue a phage rendered inactive by deletion of its endogenous SSR unit. In addition, we found in other *B. subtilis* strains and related species closely related prophages with distinct SSR units, suggesting that transfer between MGEs of independent SSR units is possible.

## Results

### SSR Units Are Functionally Exchangeable between Lysogenic Phages and ICEs

**Figure 1:**
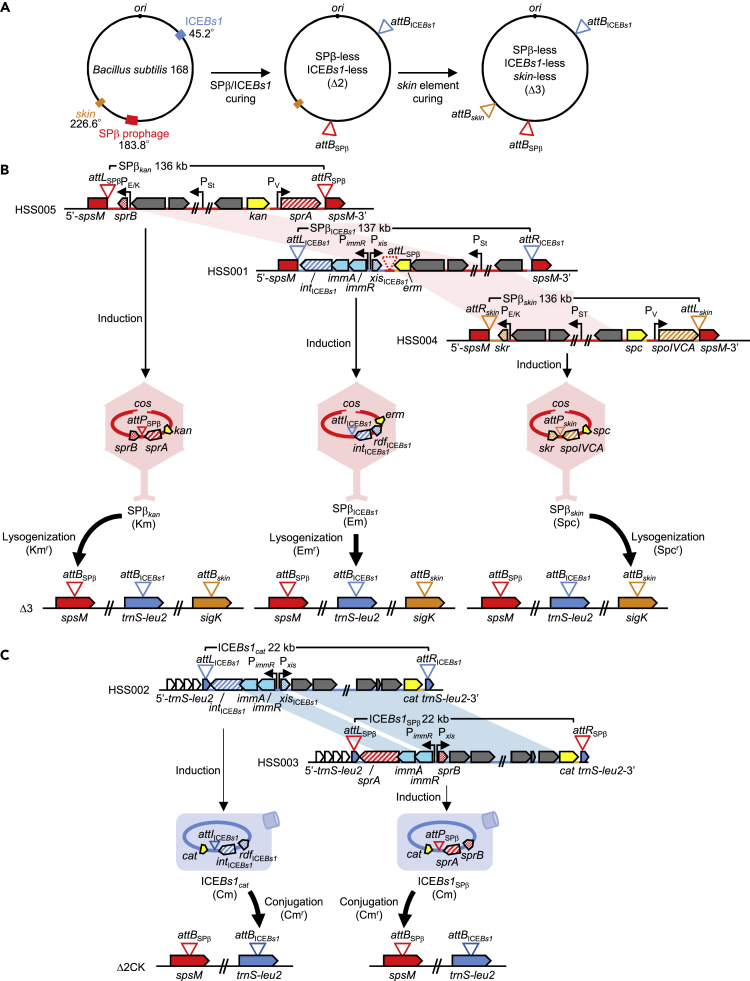
Integration of Chimeric Phages and Chimeric Integrative Conjugative Elements (ICEs) into Distinct *attB* Sequences (A) Integration sites of SPβ, ICE*Bs1*, and *skin* in the *B. subtilis* 168 genome. SPβ, ICE*Bs1*, and *skin* were cured from the *B. subtilis* 168 genome resulting in a strain (Δ3) that does not carry SPβ-, ICE*Bs1*-, and *skin*. (B) Integration of chimeric phages. The chimeric phages SPβ_ICE*Bs1*_ and SPβ_*skin*_ were constructed at *attB*_SPβ_ (the native site of SPβ), generating the HSS001 and HSS004 strains, respectively. Chimeric-phage genomes were excised following mitomycin C (MMC) treatment and packaged into phage particles. cos refers to the cohesive end sites of phage genomes. To obtain lysogens, the Δ3 strain was infected with these phages and subjected to antibiotic selection. Genomes of SPβ_ICE*Bs1*_ and SPβ_*skin*_ were integrated into *attB* sites located within *trnS-leu2* (*attB*_ICE*Bs1*_) and *sigK* (*attB*_*skin*_) genes, respectively. Red-shaded connectors represent SPβ-derived genomic regions. Horizontal black arrowheads indicate positions and directions of promoters as follows: P_V_, σ^A^-dependent promoter; P_E/K_ mother cell-specific σ^E/K^-dependent sporulation promoter; P_St_, stress inducible σ^A^-dependent promoter; P_*immR*_, σ^A^-dependent promoter of ICE*Bs1*; P_*xis*_, stress or quorum sensing-controlled promoter of ICE*Bs1*. (C) Integration of the chimeric ICE. ICE*Bs1*_SPβ_ was constructed at the native position of ICE*Bs1*, and the resulting strain was designated HSS003. The ICE genome was excised following treatment with MMC and transferred into recipient cells by conjugation. Transconjugants were obtained by selecting for chloramphenicol resistance of ICE*Bs1*_*cat*_ and ICE*Bs1*_SPβ_ and kanamycin resistance of recipient cells. After transfer to recipient cells, the ICE*Bs1*_SPβ_ genome was integrated into the *attB* site within the *spsM* gene (*attB*_SPβ_). Blue-shaded connectors represent the ICE*Bs1*-derived genomic region. Horizontal black arrowheads indicate positions and directions of transcriptional promoters. See also Figures S1 and S2 and Tables S1–S3 and S5.

**Figure 2:**
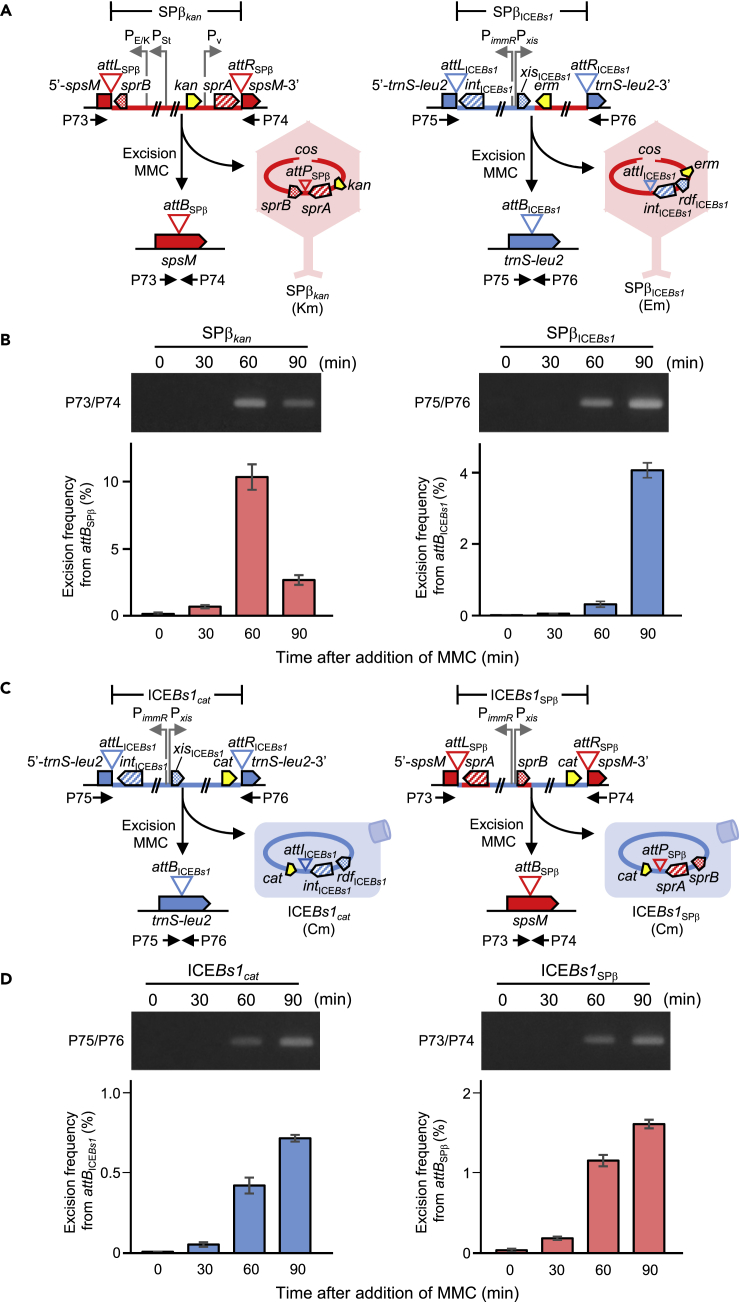
Excision of SPβ_ICE*Bs1*_ and ICE*Bs1*_SPβ_ (A) Excision mechanisms for SPβ_*kan*_ and SPβ_ICE*Bs1*_. SPβ_*kan*_ and SPβ_ICE*Bs1*_ phage excision was induced by MMC treatment. 5′-*spsM* and *spsM*-3′, as well as 5′-*trnS-leu2* and *trnS-leu2*-3′, were combined to generate *attB*_SPβ_ and *attB*_ICE*Bs1*_ in host genomes, respectively. Horizontal black arrowheads indicate the positions of primers for PCR amplification. (B) Analysis of SPβ_*kan*_ and SPβ_ICE*Bs1*_ genome excision. Total genomic DNA was extracted from MMC-treated cells. The presence of *attB* was confirmed by PCR (top panel) and quantitative PCR (qPCR) analysis (bottom panel). SPβ_*kan*_ and SPβ_ICE*Bs1*_ lysogens were grown in Luria-Bertani (LB) medium. Vegetative cells in the early log phase (OD_600_ ~ 0.2) were treated with 0.5 μg/mL MMC, and the cells were harvested at indicated times. The X axis represents time after MMC treatment in minutes. (C) Excision mechanisms for ICE*Bs1*_*cat*_ and ICE*Bs1*_SPβ_. ICE*Bs1*_*cat*_ and ICE*Bs1*_SPβ_ excisions were induced by MMC treatment. 5′-*trnS-leu2* and *trnS-leu2*-3′, as well as 5′-*spsM* and *spsM*-3′, were recombined to generate *attB*_ICE*Bs1*_ and *attB*_SPβ_ in host genomes, respectively. Horizontal black arrowheads indicate the positions of primers for PCR amplification. (D) Analysis of ICE*Bs1*_*cat*_ and ICE*Bs1*_SPβ_ genome excision. Total genomic DNAs were extracted from MMC-treated cells, and the presence of *attB* was confirmed using PCR amplification (top panel) and qPCR analysis (bottom panel). In (B) and (D), amplification of *attB*_SPβ_ (PCR and qPCR, 305 bp) and *attB*_ICE*Bs1*_ (PCR and qPCR, 497 bp) by PCR and qPCR analysis. Data are mean ± SD; n = 3 independent experiments. See also Table S4.

In the *B. subtilis* 168 genome, the 134-kb SPβ prophage (Lazarevic et al., 1999) integrates into *attB*_SPβ_ within the *spsM* gene at approximately 183.8°, whereas the 20-kb ICE*Bs1* integrates at the *attB*_ICE*Bs1*_ site within the *trnS-leu2* gene at approximately 45.2° (Figures 1A, S1A, and S1B and Table S1) (Kunst et al., 1997). SPβ and SPβ_*kan*_ (i.e., a version of the prophage modified by introduction of a kanamycin resistance cassette) carry the SSR unit *attL*_SPβ_-*sprB*-*sprA*-*attR*_SPβ_. Among the components of this unit, the integrase gene, *sprA*, encodes an LSR and has a σ^A^-dependent promoter (P_V_), whereas its cognate RDF gene, *sprB*, has distinct promoters, i.e., a stress (mitomycin C, MMC)-inducible σ^A^-dependent promoter (P_St_) and a sporulation-specific σ^E/K^-dependent promoter (P_E/K_) (Figure 1B) (Abe et al., 2014). ICE*Bs1* and ICE*Bs1*_*cat*_ (a version carrying a chloramphenicol resistance cassette) harbor the SSR unit *attL*_ICE*Bs1*_-*int*_ICE*Bs1*_-*xis*_ICE*Bs1*_-*attR*_ICE*Bs1*_ (Tables S1 and S2) (Lee et al., 2007). The integrase gene *int*_ICE*Bs1*_ encodes a Tyr-type recombinase and is constitutively expressed with *immA* and *immR* from the P_*immR*_ promoter. In contrast, the cognate RDF gene *xis*_ICE*Bs1*_ is expressed from the P_*xis*_ promoter, which is regulated by SOS responses and quorum sensing signals mediated by ImmA (anti-repressor) and ImmR (immunity repressor) (Figure 1C) (Auchtung et al., 2005, Auchtung et al., 2007, Lee et al., 2007, Bose et al., 2008). We investigated whether the ICE*Bs1*-derived SSR unit would function in the context of SPβ insertion and excision. First, we determined whether a chimeric SPβ_ICE*Bs1*_ construct would integrate preferentially into the *attB*_ICE*Bs1*_ site or the *attB*_SPβ_ site. The 137-kb chimeric SPβ_ICE*Bs1*_ prophage was generated by replacing the SPβ-derived SSR unit (*attL*_SPβ_-*sprA*-*sprB*-*attR*_SPβ_) with the ICE*Bs1-*derived SSR unit (*attL*_ICE*Bs1*_-*int*_ICE*Bs1*_-*xis*_ICE*Bs1*_-*attR*_ICE*Bs1*_) (Figure 1B) and introduction of an erythromycin resistance cassette. In this construct, *int*_ICE*Bs1*_ and *xis*_ICE*Bs1*_ remained under the control of ICE*Bs1*-derived P_*immR*_ and P_*xis*_ promoters, respectively, and, as expected, were expressed upon integration/excision of ICE*Bs1*. The resulting SPβ_ICE*Bs1*_ chimeric-phage lysogen (HSS001) was induced by MMC (0.5 μg/mL) and the phage lysate was used to infect a SPβ-, ICE*Bs1*-, and *skin*-cured strain (Δ3) (Figure 1B). SPβ_ICE*Bs1*_ lysogens were then selected for erythromycin resistance, and the sequences of the regions flanking *attL*_ICE*Bs1*_ and *attR*_ICE*Bs1*_ were determined. These analyses showed integration of SPβ_ICE*Bs1*_ at *attB*_ICE*Bs1*_ in the genome of the Δ3 strain (Figure S2A). Next, to detect phage excision and the regeneration of the *attB* and *attP* sites from the *attL* and *attR* junctions of the lysogen, we performed polymerase chain reaction- (PCR) and quantitative polymerase chain reaction (qPCR)-based analyses. These assays showed that, although the timing of SPβ_ICE*Bs1*_ excision was slower than that of SPβ_*kan*_ because of transcriptional regulation of SSR unit in SPβ_ICE*Bs1*_, excision rates were significantly increased in response to MMC-mediated induction for both the SPβ_*kan*_ and chimeric SPβ_ICE*Bs1*_ lysogens (Figures 2A and 2B). SPβ_ICE*Bs1*_ excision was also measured by counting plaque-forming units/mL (pfu/mL). Phage titers for SPβ_ICE*Bs1*_ were found to be slightly lower than those of the positive controls, i.e., wild-type SPβ or SPβ_*kan*_ (Table 1). Even though the integration rate for SPβ_ICE*Bs1*_ was approximately 30-fold lower than that of SPβ_*kan*_ (probably due to non-native SSR units), site-specific integration at *attB*_ICE*Bs1*_ was 100% accurate in lysogens carrying SPβ_ICE*Bs1*_ (i.e., there was no integration at the *attB*_SPβ_ site). In total, these data indicate that the ICE*Bs1-*derived SSR units are sufficient for integration/excision of SPβ at the *attB*_ICE*Bs1*_ site. These results further imply that SSR units can be repurposed to control the life cycle of unrelated MGEs, including that of lysogenic phages.

**Table 1:** Phage Titration and Lysogenic Frequency

Phages	Phage Titer (pfu)^a^	Integration Frequency^a^^,^^b^	Integrated at *attB* Sites (%)^c^
SPβ	1.3 (±0.8) × 10^8^	–	–
SPβ_*kan*_	8.4 (±2.8) × 10^7^	4.2 (±3.2) × 10^−4^	100
SPβ_*skin*_	1.2 (±0.5) × 10^8^	8.4 (±2.3) × 10^−4^	100
SPβ_ICE*Bs1*_	4.1 (±0.9) × 10^7^	1.4 (±1.0) × 10^−5^	100

a The data shown are the average of three independent experiments ±SD.

b Infected by MOI = 0.1

c Twenty lysogens were investigated.

Our next objective was to investigate whether the SPβ-derived SSR unit was similarly adaptable for use in ICE*Bs1.* We engineered a 22-kb chimeric ICE*Bs1*_SPβ_ carrying a chloramphenicol resistance cassette and the SPβ-derived SSR unit instead of the ICE*Bs1*-derived SSR unit (Figure 1C and Tables S1 and S2). In this construct, SPβ-derived *sprA* and *sprB* genes were initially placed under the control of P_*immR*_ and P_*xis*_ promoters, respectively. The resulting chimeric ICE was designated ICE*Bs1*_SPβN_, yet excision of ICE*Bs1*_SPβΝ_ was detected even in the absence of MMC-mediated induction. Expression of *sprB* under these conditions could be due to the leakiness of the P_*xis*_ promoter combined with the strong Shine-Dalgarno (SD) sequence of *xis*_ICE*Bs1*_. To reduce *sprB* expression, we introduced a GGAGG to GcAGG mutation into the SD sequence, 11 bp upstream of the start codon (TTG) of *xis*_ICE*Bs1*_. This second chimeric ICE construct (designated as ICE*Bs1*_SPβ_) was stably integrated into *attB*_SPβ_ under non-MMC treatment conditions and was excised following addition of MMC (0.5 μg/mL). We performed PCR and qPCR-based detection assays and showed that the excision rates of both ICE*Bs1*_SPβ_ and the parent ICE*Bs1*_*cat*_ were increased in response to MMC-mediated induction (Figures 2C and 2D). These observations indicate that the SPβ-derived SSR unit, *attL*_SPβ_-*sprB*-*sprA*-*attR*_SPβ_, is functional in the context of ICE*Bs1* integration and excision. Next, the strain harboring ICE*Bs1*_SPβ_ was induced by addition of MMC and co-cultured with the Δ2CK strain. That strain had been cured of SPβ and ICE*Bs1*, and natural transformation was prevented by disruption with a kanamycin resistance cassette of the major competence gene *comK*. We then selected for ICE*Bs1*_SPβ_ Δ2CK strains based on acquisition of chloramphenicol resistance and maintenance of kanamycin resistance. ICE*Bs1*_SPβ_ transconjugants were obtained with approximately the same frequency as parental-type ICE*Bs1*_*cat*_ (Table S3). DNA sequencing of the flanking region of *attL*_SPβ_ and *attR*_SPβ_ confirmed that ICE*Bs1*_SPβ_ had integrated at *attB*_SPβ_ in the Δ2CK strain, and site-specific integration of ICE*Bs1*_SPβ_ at *attB*_SPβ_ had 100% accuracy (Figure S2B and Table S3). Hence, the SPβ-derived SSR unit is sufficient to drive SSR of ICE*Bs1*. Taken together, these data indicate compatibility of SSR units between the prophage and the ICE.

### The SSR Unit Derived from the Defective Prophage *Skin* Is Active when Inserted into a Modified SPβ Prophage

**Figure 3:**
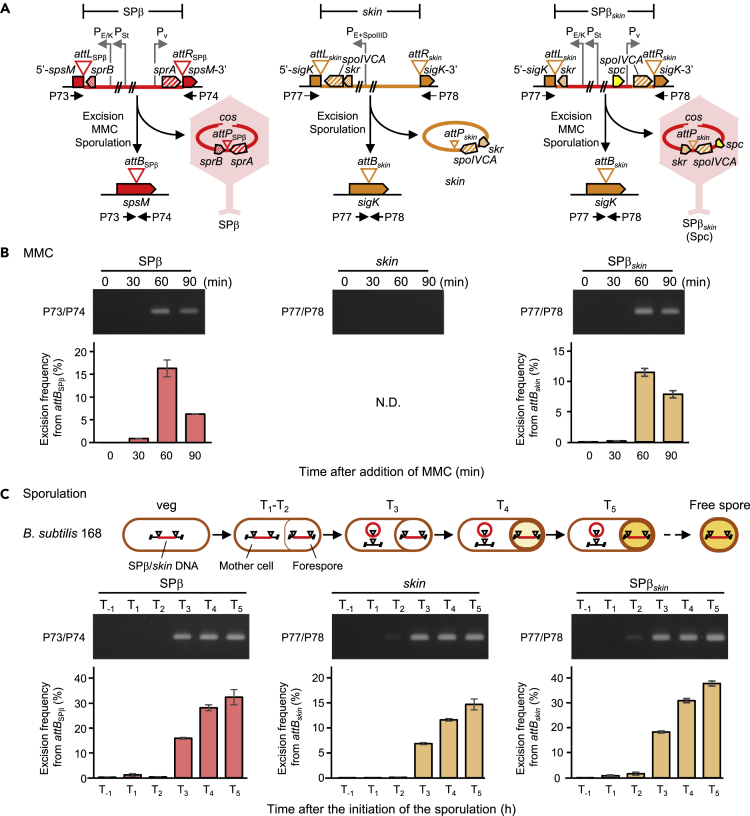
Excision of SPβ_*skin*_ Using Site-Specific Recombination (SSR) Units from a Defective Prophage (A) Excision mechanisms for SPβ, *skin*, and SPβ_*skin*_ (from left to right). SPβ and SPβ_*skin*_ excisions were induced either after MMC treatment or during sporulation, whereas *skin* excision was observed only during sporulation, as previously reported (Kimura et al., 2010); Pv, σ^A^-dependent promoter; P_St_, stress-inducible promoter; P_E/K_, mother cell-specific σ^E/K^-dependent sporulation promoter; P_E+SpoIIID_, mother cell-specific σ^E^ and SpoIIID-dependent sporulation promoter. Horizontal black arrowheads indicate the positions of primers for PCR amplification. (B) Excision in the presence of MMC. SPβ and SPβ_*skin*_ genomes were excised from host genomes after MMC treatment, whereas the defective prophage *skin* (no stress-inducible promoter) was not. *B subtilis* 168 cells containing SPβ, *skin*, or SPβ_*skin*_ lysogens were grown in LB medium. Vegetative cells in the early log phase (OD_600_ ~ 0.2) were treated with 0.5 μg/mL MMC and were harvested at indicated times and analyzed by PCR amplification (top panel) and qPCR (bottom panel). N.D., not detected. (C) Excision during sporulation. Schematics of SPβ and *skin* excision during sporulation are shown above figure. *B. subtilis* 168 sporulating cells divided asymmetrically to produce mother cells and forespores at 1–2 h after the initiation of sporulation (T_1_–T_2_). Subsequently, *skin* and SPβ excision was specifically induced in mother cells at approximately 3 h after the initiation of sporulation (T_3_) (Abe et al., 2014, Sato et al., 1990). *B. subtilis* 168 cells and SPβ_*skin*_ lysogens were grown in DSM, and samples of vegetative cells were collected (OD_600_ ~ 0.2; T_−1_) at indicated times at 1-h intervals (T_1_–T_5_). Middle and bottom panels represent PCR amplification and qPCR analysis, respectively. In (B) and (C), Amplification of *attB*_SPβ_ (PCR and qPCR, 305 bp) and *attB*_*skin*_ (PCR and qPCR, 221 bp) by PCR and qPCR analysis. Data are mean ± SD; n = 3 independent experiments. See also Figure S3 and Table S4.

The *sigK* intervening *skin* element located at approximately 226.6° also carries an SSR unit (*attL*_*skin*_, *int*_*skin*_ = *spoIVCA*, *attR*_*skin*_). The integrase gene *spoIVCA* encodes an LSR that catalyzes the joining of the truncated 5′-*sigK* and *sigK*-3′ portions of the *sigK* gene (Sato et al., 1990, Kunkel et al., 1991) (Figures 1A and 3A). Although *spoIVCA* is well characterized, the cognate *rdf* gene remains unidentified. To identify *rdf* in the *skin* element, we performed deletion analyses in the region flanking *spoIVCA*, based on the observation that in other systems the *rdf* gene is usually located near the integrase gene (for instance, *int-xis* in phage λ). Because the promoter of *spoIVCA* (Pσ^E^) is located over 200 bp upstream of the translational start site, we hypothesized that an open reading frame (ORF) was present immediately upstream of the *spoIVCA* coding sequence. To confirm the presence of the ORF and its role as the *rdf* of *skin,* we introduced the IPTG-inducible P*spac* promoter at positions +47, +18, and −17 nucleotides (nt) from the first nucleotide of the putative ORF (Figures S3A and S3B). We observed that the excision of *skin* was only induced following expression from the −17 nt position (Figures S3C and S3D). We named this small ORF *skr*. It encodes a 64-amino-acid (aa) protein and is required for the reconstitution of a full *sigK* gene from the 5′-*sigK* and *sigK*-3′ portions. Unexpectedly, the 3′-end of *skr* overlaps with the 5′-end of *spoIVCA* by 101 nt, owing to a +1 frameshift (Figure S3A).

Next, we constructed a 136-kb chimeric SPβ_*skin*_ prophage that carries the *skin* SSR unit *attL*_*skin*_-*skr*-*spoIVCA*-*attR*_*skin*_ and a spectinomycin resistance cassette (Figure 1B and Tables S1 and S2). In this chimeric prophage, *spoIVCA* and *skr* transcription levels are controlled by P_V_ of *sprA* and both the P_St_ and P_E/K_ promoters of *sprB*, whereas the SSR unit of SPβ (*attL*_SPβ_-*sprB*-*sprA*-*attR*_SPβ_) was eliminated. The resulting lysogen contained the chimeric SPβ_*skin*_ prophage. Upon treatment with MMC, the phage lysate was used to infect the Δ3 host strain and the SPβ_*skin*_ lysogen was selected for spectinomycin resistance. DNA sequences of the flanking regions of *attL*_*skin*_ and *attR*_*skin*_ were then determined to confirm that SPβ_*skin*_ was integrated at *attB*_*skin*_ in the Δ3 strain genome (Figure S2C). Phage titers were determined (pfu/mL) on a lawn of Δ3 strain cells (Table 1). Excision of SPβ_*skin*_ from *attB*_*skin*_ after MMC addition was quantified by PCR and qPCR (Figure 3B). The integration frequency of SPβ_*skin*_ was comparable with that of SPβ_*kan*_, and the accuracy of site-specific integration at *attB*_*skin*_ was 100% (Table 1). As mentioned before, excision of *skin* and SPβ both occur during sporulation (Figure 3C), leading to reconstitution in the mother cell genome of the *sigK* and *spsM* genes, respectively. Similarly, SPβ_*skin*_ excision was observed 3 h after initiation of sporulation (T_3_). Rearrangement of *sigK* was as accurate as *skin* (Figure 3C). These results indicate that the integration system of SPβ can replace that of *skin* and that the *skin*-derived SSR unit can also drive the excision of the active lysogenic phage SPβ, even though it is derived from a defective prophage.

### Diversity of Prophage Genomes Integrated at Specific *attB* Sites

Searching the microbial genomes database at NCBI (http://www.ncbi.nlm.nih.gov), we found that 16 *B. subtilis* strains had SPβ phage-like sequences inserted into the *spsM* gene. Each sequence was of similar size (131–134 kb) and gene organization was highly conserved (Figure S4), suggesting that they all function as lysogenic phages. In contrast, in the *B. amyloliquefaciens* group, the phage-like sequences inserted into *spsM* were much shorter (from 4 to 20 kb), indicating that they are unlikely to be active prophages (Abe et al., 2014) (Figure S5). Outside of the *B. subtilis* and *B. amyloliquefaciens* groups, no phage-like sequences were found inserted in *spsM*.

**Figure 4:**
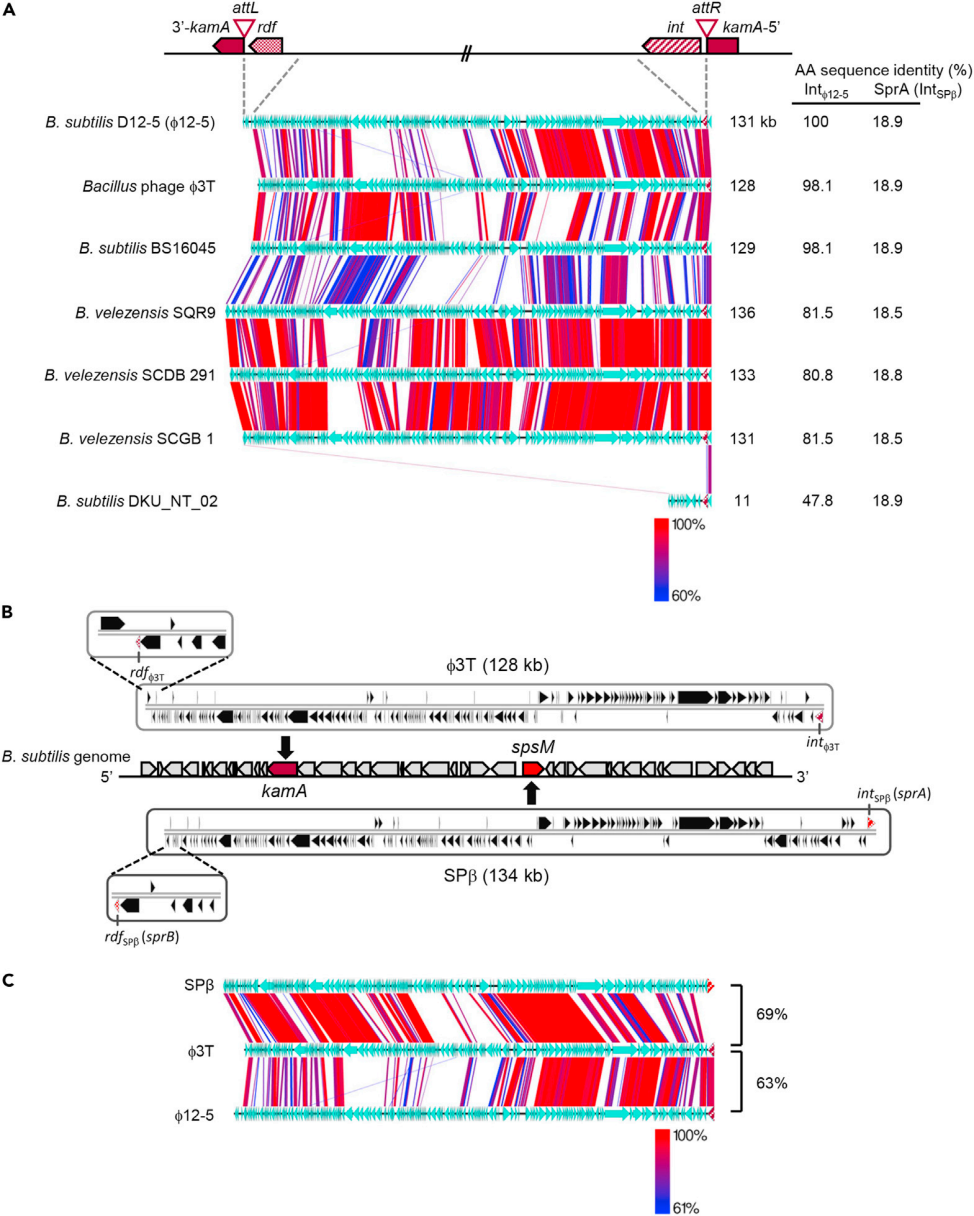
Gene Organization of SPβ-Related Phages Inserted into the *kamA* Gene (A) Synteny of SPβ-related phages residing in *kamA*; the top diagram shows the positions of the integrase (*int*) and putative recombination directionality factor (*rdf*) genes. Host strains and phage names are indicated on the left of the diagram and lengths on the right. Integrase amino acid sequence homologies (%) to Int_φ12-5_ or SprA (Int_SPβ_) are shown on the right column. (B) Comparisons between the SPβ and φ3T phage genomes. The enlarged views on the left-hand side show the genes flanking the *rdf*. Vertical black arrowheads indicate the position of the integration site. (C) Synteny of the SPβ, φ3T, and φ12-5 genomes. Genome data were extracted from the genome database at the national center for biotechnology information (NCBI). Sequence comparisons were performed using tBLASTx; red-blue lines indicate regions with 60%–100% identity. Genome alignment figures were created using Easyfig (Easyfig 2.2.2 for generating tBLASTx alignment files and visualization). Genetic homology (tBLASTx) percentages are included on the right of the diagram. See also Figures S4 and S5 and Table S2.

In *B. subtilis* strain D12-5, we noticed a prophage named φ12-5, whose genome organization closely resembled that of SPβ (Figure 4A). φ12-5 disrupted the *kamA* gene, which was previously reported to be a sporulation gene expressed in the mother cell under the control of σ^E^ (Eichenberger et al., 2003, Eichenberger et al., 2004, Feucht et al., 2003). In *B. subtilis* 168, this gene is annotated as coding for an L-lysine 2,3-aminomutase. Interestingly, *kamA* (formerly *yodO*) is in the vicinity of *spsM* (formerly *yodU*), with just a few genes separating the two phage insertion sites (Figure 4B). Although the genome organization of φ12-5 is conserved in comparison with SPβ, its SSR unit is different. Homology searches (blastp) using the integrase (Int_φ12-5_) of strain D12-5 as query revealed that seven phage-like (128–136 kb) and one non-phage-like (11 kb) sequences are inserted into *kamA* in various *Bacillus* species (Figure 4A). Among these, φ3T of *B. subtilis* reportedly (Erez et al., 2017, Dou et al., 2018) functions as a vital prophage. Moreover, the integrase Int_φ3T_ (displaying 18.9% identity with SprA of SPβ) and its cognate RDF, Rdf_φ3T_ (no similarity with SprB of SPβ), were encoded by genes located in the flanking regions of the φ3T prophage (Figures 4A and 4B). A degenerate phage-sequence (11 kb) disrupting *kamA* was found in only one *B. subtilis* strain, DKU_NT_02. Among the *kamA*-inserted prophages in other strains, diverse gene organizations were observed. Furthermore, genome organizations of φ3T and φ12-5 (both inserted into *kamA*) were only 63% similar (comparison percentage generated using tBLASTx), whereas those of SPβ (*spsM*) and φ3T (*kamA*) were 69% similar. Thus, the degree of genomic similarity between prophages containing heterogeneous SSR units (SPβ versus φ3T) is higher than that between homogeneous SSR units (φ3T versus φ12-5) (Figure 4C). The presence of heterogeneous SSR units in similar prophages (SPβ and φ3T) suggests that the correspondence between prophages and their cognate SSR units is not absolute.

### The *kamA* Gene Is Reconstituted during Sporulation by Excision of the SPβ-like Phage φ3T

**Figure 5:**
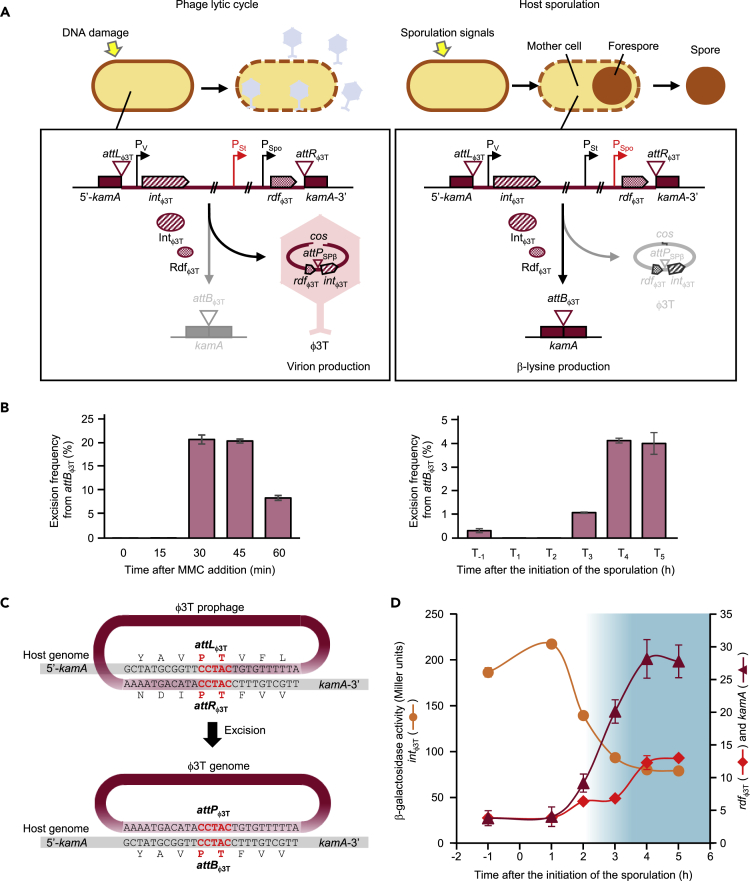
DNA Rearrangement of *kamA* in the φ3T Lysogen (A) Diagram of φ3T prophage excision and *kamA* rearrangement. During the lytic cycle, excised φ3 transfer DNA is packaged into phage capsids to produce virions and promote host cell lysis. During sporulation, prophage excision generates a functional *kamA* gene. Horizontal arrowheads indicate positions and directions of putative promoters, and red arrowheads represent the active promoter of *rdf*_φ3T_; P_V_, vegetative promoter; P_spo_, sporulation-specific promoter; P_St_, stress-inducible promoter. (B) Excision of φ3T; bar graphs show qPCR analyses of *attB*_φ3T_ (229-bp) generated by φ3T excision upon MMC treatment (left) and during sporulation (right). (C) Nucleotide sequences of φ3T attachment sites before and after genome excision; the 5-bp long overlapping nucleotide sequence is indicated with red letters. Translated amino acid sequences are shown above or below nucleotide sequences. (D) β-Galactosidase activity of *int*_φ3T_–, *rdf*_φ3T_–, and *kamA*–*lacZ* reporter constructs during sporulation; *int*_φ3T_, *rdf*_φ3T_, and *kamA* genes were transcriptionally fused to the *lacZ* reporter gene in ESI-φ3T, ESR-φ3T, and INDkamA, respectively. φ3T excision occurred at 3 h after the initiation of sporulation (T_3_; blue-shaded areas). In (B) and (D), data are mean ± SD; n = 3 independent experiments. See also Figures S1, S6, and S7 and Tables S2 and S5.

Previous studies have shown that phage φ3T is integrated between positions 2,106,060 and 2,106,064 in the *B. subtilis* BEST7003 genome (Goldfarb et al., 2015). This site (i.e., the putative DNA breakpoint for integration of φ3T) corresponds to a CCTAC sequence in the *kamA* gene. The N- and C-terminal encoding portions of *kamA* were named 5′-*kamA* and *kamA*-3′, respectively (Figure 4A). Imperfect inverted repeat sequences (23 24 bp long) were found adjacent to the CCTAC site and may provide binding sites for a site-specific recombinase (Figure S1D). As mentioned above, *kamA* is a member of the σ^E^ regulon (Feucht et al., 2003, Eichenberger et al., 2003, Eichenberger et al., 2004), suggesting that φ3T is excised during sporulation to reconstitute the composite *kamA* gene in the mother cell genome. We confirmed that φ3T was excised from *attL*_φ3T_ and *attR*_φ3T_ sites upon MMC treatment and also during sporulation (Figures 5A and 5B). When the φ3T lysogen was treated with MMC, φ3T was excised from the *kamA* gene after 30 min (Figure 5B). We confirmed φ3T excision during sporulation by analyzing DNA samples from sporulating φ3T lysogens. In these experiments, φ3T was excised at hour 3 of sporulation in the absence of MMC (Figures 5B and S6A). Next, we sequenced and identified the flanking sequences at the junctions (*attL*_φ3T_ and *attR*_φ3T_). Because the *attP*_φ3T_ sequence is conserved between *attL*_φ3T_ and *attR*_φ3T_, its presence was determined by comparison of DNA sequences before (*attL*_φ3T_ and *attR*_φ3T_) and after (*attB*_φ3T_) excision of φ3T. These analyses showed that φ3T excision combines the 5′-*kamA* and *kamA*-3′ in frame during sporulation (Figure 5C). We then identified an *int* gene and its cognate *rdf* gene as components of the SSR unit of φ3T (*attL*_φ3T_-*rdf*_φ3T_-*int*_φ3T_-*attR*_φ3T_) by replacing the native promoter of each gene with the IPTG-inducible promoter P*spac*. In both *int*_φ3T_- (ESI-φ3T) and *rdf*_φ3T_- (ESR-φ3T) inducible strains, no excision was detected in the absence of IPTG, neither following MMC treatment nor during sporulation (Figures S6B and S6C). But in the presence of IPTG, the excision pattern of ESI-φ3T strains was similar to that of the wild-type φ3T lysogen, whereas excision in ESR-φ3T strains was detected regardless of induction of sporulation or SOS response (via addition of MMC). In agreement, growth inhibition was observed only in ESR-φ3T (Figure S6D). These data suggest that the *erm* gene is lost upon addition of IPTG, because the *rdf*_φ3T_ gene was induced, thus producing the RDF that regulates φ3T prophage excision. Next, we examined the expression of *int*_φ3T_, *rdf*_φ3T_, and *kamA* from *lacZ* fusion constructs. Strains harboring *int*_φ3T_*-lacZ* (in ESI-φ3T), *rdf*_φ3T_*-lacZ* (in ESR-φ3T), and *kamA-lacZ* (in INDkamA) were constructed and analyzed. During vegetative growth and sporulation, *int*_φ3T_*-lacZ* was constitutively expressed. Yet, *rdf*_φ3T_*-lacZ* and *kamA-lacZ* were expressed concomitantly 2 h after the initiation of sporulation, indicating that *rdf*_φ3T_ controls the timing of φ3T excision (Figures 5D and S7). Collectively, these data demonstrate that the φ3T prophage, which is highly similar to the SPβ prophage except for its SSR units, regulates *kamA* expression by excision of the φ3T prophage during sporulation. Even though the SPβ and φ3T prophages were highly similar, they integrated into specific *attB* sites. Importantly, the specificity of integration depended solely on the nature of the corresponding SSR unit. These findings are consistent with the hypothesis that SSR units are adaptable between MGEs.

## Discussion

**Figure 6:**
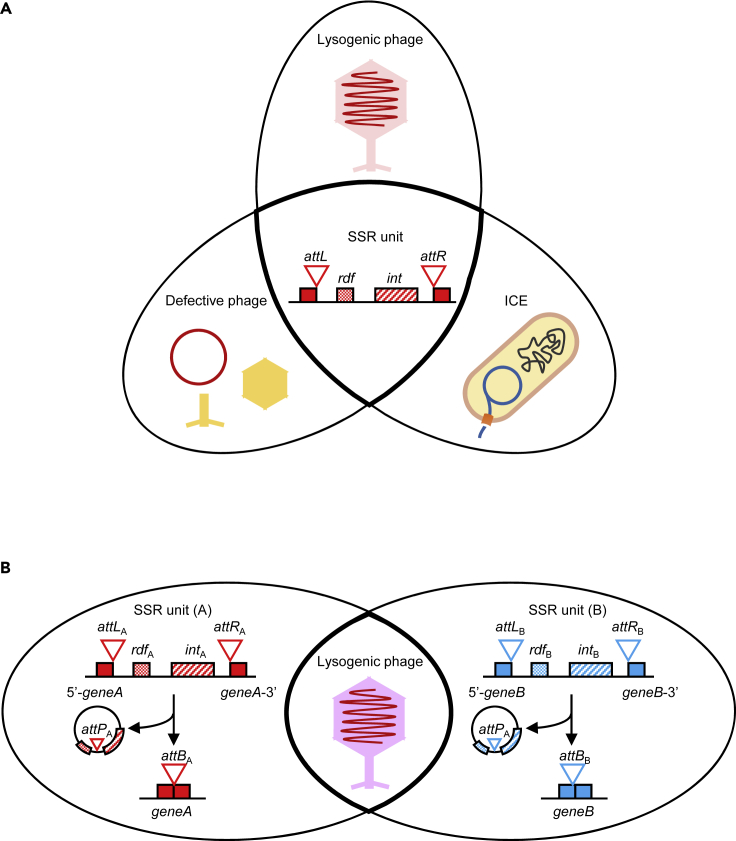
A Model for the Compatibility of SSR Unit in MGEs (A) Compatibility of SSR units in MGEs. SSR units are functionally exchangeable between lysogenic phages, defective prophages, and ICEs. (B) Homologous phages existing in different SSR units. In this study we showed the clues to the conversion of SSR unit in homologous lysogenic phages (e.g., A:SPβ and B:φ3T) in the nature.

In bacteria, mobilization of lysogenic phages and ICEs consists of a series of successive steps, starting with excision, followed by intercellular transfer and finally integration of the genetic material into new host cells. Excision and integration are mediated by SSR units (*attL-int-rdf-attR*). Therefore, acquisition of individual SSR units is a key factor driving MGE evolution. Many different types of SSR units have been found in bacterial genomes. Each SSR unit carries a gene encoding an individual member of the integrase family (either a Tyr- or a Ser-type enzyme) that recognizes cognate *attP* and *attB* sites with high selectivity. Some site-specific recombination systems, including P1 (Cre), Bxb1, TP901-1, R4, and φC31 integrases, have been widely used as tools to introduce foreign genes, carried, for instance, on site-specific integration plasmids, in a range of organisms, including other microbes, plants, and mammalian cells (Hirano et al., 2011, Fogg et al., 2014, Meinke et al., 2016). In this study, we demonstrate that, after artificial exchange of SSR units between a lysogenic phage and an ICE, these units remain functional and specific to the *attB* site recognized by their respective integrase. Specifically, SPβ and ICE*Bs1* are two MGEs in *B. subtilis* that recognize different *attB* sites based on the SSR unit they carry. Yet, these SSR units are not restricted to their MGE; on the contrary, they are interchangeable and remain fully functional when inserted into other lysogenic phages and ICEs (Figure 6A). Only slight reductions in phage titers and integration frequencies were observed with non-native SSR units. This in contrast with other phage elements, like tails or capsids. In a recent study, it was demonstrated that the exchange of phage tails altered the host ranges (Ando et al., 2015). Although capsids or tails can be exchanged from phage to phage, adaptation to unrelated virion proteins is an issue. Since the SSR unit is not a structural part of the virion, distinct SSR units that vary in their recognition sites (*attB*) can be viewed as highly adaptable phage components. By extension, adaptability of SSR units may constitute an important factor modulating plasticity among MGEs in their interaction with host genomes.

To allow adaptation of SSR units to a new MGE, the corresponding *int* and *rdf* genes must be expressed at appropriate times in the host life cycle (Ghosh et al., 2006, Fogg et al., 2014, Merrick et al., 2018). Considering that *int* is required for both integration and excision, *int* genes are often constitutively expressed. In contrast, regulation of RDF production is more elaborate, presumably because the role of RDF is limited to excision. Furthermore, because excision often occurs early in the lytic cycle, *rdf* is often among the first transcription units induced by SOS responses (Khaleel et al., 2011, Jain and Hatfull, 2000, Ghosh et al., 2006). In addition to regulating prophage excision, these responses also condition ICE transfer. In *B. subtilis*, the *immR*-*immA* operon is the regulatory module integrating SOS response and cell density signals to promote the mobilization of ICE*Bs1*. Although *immR*-*immA* is constitutively transcribed (along with the downstream gene *int*_ICE*Bs1*_), expression of the RDF (*xis*_ICE*Bs1*_) is repressed by ImmR, whose degradation by ImmA is dependent on SOS and cell density signals (Auchtung et al., 2005, Auchtung et al., 2007, Lee et al., 2007, Bose et al., 2008). In the present study, we had to place *sprB* (the gene encoding the RDF of SPβ) immediately downstream of P_*xis*_ to ensure control by the *immR-immA* system. As a result, excision of the chimeric SPβ_ICE*Bs1*_ prophage in the lysogen was detected following addition of MMC and induction of the SOS response, thus showing that regulation by the *immR-immA* module can be co-opted for prophage excision. This result agrees with a previous report that the *B. subtilis* lysogenic phage φ105 relied on a similar *immR-immA* system (Bose et al., 2008). Thus, at least some induction systems regulated by RDFs are common among MGEs, regardless of prophage or ICE origin.

Other lysogenic phages containing SSR units similar to those of SPβ and φ3T were found to be present in multiple organisms, especially variations on the basic SSR unit: *attL*, *int, rdf*, and *attR*. As shown in Figure 4B, in the SPβ and φ3T prophages, *int* and *rdf* are located at both ends of the element (close to *attL* or *attR*). After excision, however, *int* and *rdf* are only separated by the *attP* locus. In this circular genome state, RecA-mediated homologous recombination could promote exchange of *int-attP-rdf* cassettes between MGEs, especially between circular phage genomes. The presence of several lysogenic phages (SPβ and φ3T) in a single strain further supports our hypothesis that SSR units are transferable between MGEs (Figure 6B). One advantage of having lysogenic phages with different SSR units may be that prophages could gain the ability to integrate into other *attB* sites in the host genome.

When certain genes, like those encoding restriction enzymes, methyltransferases, toxin-antitoxin modules, or drug resistance enzymes, are carried by an MGE, they may influence the stability of that element. A key factor favoring maintenance of SSR units in intervening elements interrupting sporulation genes is that gene reconstitution is necessary for survival through sporulation. This might constitute an even larger evolutionary advantage than prophage ability to excise in response to the SOS system. This could explain why most of the small intervening elements in sporulation genes are no longer functional prophages but are maintained in the host genome because of the role they play in gene reconstitution (Figure S5). SSR units that split sporulation genes act to rejoin interrupted genes specifically during sporulation, and the genes are usually dispensable during growth. However, mutations that prevent reconstitution of the interrupted gene into a functional gene during sporulation would likely be eliminated by natural selection, because of the survival advantage provided by the ability to sporulate. Furthermore, it should be noted that almost all intervening elements in sporulation genes were integrated into mother cell-specific genes (Abe et al., 2013, Abe et al., 2014, Abe et al., 2017a). Mother cells are killed by lysis at the end of sporulation, whereas the intact spore genome is protected in a highly resistant dormant cell, thus the intervening element is maintained in its genome. For all these reasons, SSR units represent an advantageous platform for functional lysogenicity and are especially favored in spore-forming bacteria. In this report, we also showed that the SSR unit of the defective prophage *skin* becomes active after introduction into the SPβ genome (SPβ_*skin*_). Thus, although the degradation of a prophage causes defectiveness, defective prophages can turn back into lysogenic phages by addition of SSR units (and possibly adjacent genes) under natural conditions (Figure 6A). Through this mechanism, phages can reacquire lysogen function by coordinating certain SSR units, implying that SSR units from lysogenic phages and ICEs share a common foundation.

We also identified an RDF gene called *skr* in the *skin* element and found that its 3′ half overlapped with the 5′ end of *spoIVCA* encoding the N-terminal region of Int_*skin*_. We have not yet determined how the +1 shift in reading frame regulated expression of *skr-spoIVCA* during sporulation and the consequences on Skr activity. A similar gene encoding an RDF from an intervening sequence interrupting *sigK* has been identified in *Clostridioides difficile* (Serrano et al., 2016). However, the SSR units of the two *skin* elements differ between *B. subtilis* and *C. difficile*, and although both recognize *attB* sites, the corresponding sequences are located in different regions of the *sigK* gene.

Finally, we characterized the SSR unit of φ3T that recognized an *attB* site in *kamA*, a composite gene that is also reconstituted during sporulation. The *kamA* gene encodes an L-lysine 2,3-aminomutase (Chen et al., 2000), an enzyme that converts L-lysine to L-β-lysine, which is the first metabolite of the lysine degradation pathway in *Bacillus* sp (Zhang et al., 2014). In *Escherichia coli*, post-translational modification by β-lysylation is required for the activity of elongation factor P (EF-P) (Park et al., 2012). We have not yet determined the precise role of β-lysylation in *B. subtilis*, but considering that *kamA* expression is controlled by the mother cell-specific sigma factor σ^E^, it may play a role in sporulation, even though deletion of *kamA* does not appear to significantly impair sporulation (Eichenberger et al., 2003).

### Limitations of the Study

Although we identified closely related prophages with distinct SSR unit, such ICEs were not found in genomic databases. These data were results based on the basic local alignment search tool (BLAST) analysis utilizing the database from the national center for biotechnology information (NCBI). To clarify the detailed SSR unit distribution, more advanced bioinformatics approaches would be required.

## Methods

All methods can be found in the accompanying Transparent Methods supplemental file.

## Supplemental Information

Document S1. Transparent Methods, Figures S1–S7, and Tables S1–S3 and S5

Table S4. Primers Used in this Study, Related to Figures 2, 3, and Transparent Methods
