# Supplementary material for: Compatibility of Site-Specific Recombination Units between Mobile Genetic Elements
Source: iScience. 2019 Dec 26;23(1):100805. doi: 10.1016/j.isci.2019.100805 (PMC6957869; doi:10.1016/j.isci.2019.100805)
Supplement: Document S1. Transparent Methods, Figures S1–S7, and Tables S1–S3 and S5 [file mmc1.pdf]

ISCI, Volume *23*

Supplemental Information

Compatibility of Site-Specific

Recombination Units

between Mobile Genetic Elements

Shota Suzuki, Miki Yoshikawa, Daisuke Imamura, Kimihiro Abe, Patrick Eichenberger, and Tsutomu Sato

A

attP$_{SP\beta}$

gtttccactaagaaaagtagtaagtatcttaaaaa**ACAGATAAAGCTGTAT**attaagatacttactacatatctaacgaaaaaa

attB$_{SP\beta}$

TTTCACACCGAGTGCAGCATGTCATTAATATCAGT**ACAGATAAAGCTGTAT**CTCCTGTGAACACAATGGGTGCCACCAAGCTGT

attL$_{SP\beta}$ (5'-*spsM*)

TTTCACACCGAGTGCAGCATGTCATTAATATCAGT**ACAGATAAAGCTGTAT**attaagatacttactacatatctaacgaaaaaa

attR$_{SP\beta}$ (*spsM*-3')

gtttccactaagaaaagtagtaagtatcttaaaaa**ACAGATAAAGCTGTAT**CTCCTGTGAACACAATGGGTGCCACCAAGCTGT

B

attP$_{ICE\textit{Bs1}}$

gtaaataaagatatgattttactagg**TTGAGGG**cctagtgggtgaataacccgtggaggttcaagtcctctcggccgcatcaat

attB$_{ICE\textit{Bs1}}$

CGTGGCGGAATGGCAGACGCGCTAGG**TTGAGGG**CCTAGTGGGTGAATAACCCGTGGAGGTTCAAGTCCTCTCGGCCGCATCTCA

attL$_{ICE\textit{Bs1}}$ (5'-*trnS-leu2*)

CGTGGCGGAATGGCAGACGCGCTAGG**TTGAGGG**cctagtgggtgaataacccgtggaggttcaagtcctctcggccgcatcaat

attR$_{ICE\textit{Bs1}}$ (*trnS-leu2*-3')

gtaaataaagatatgattttactagg**TTGAGGG**CCTAGTGGGTGAATAACCCGTGGAGGTTCAAGTCCTCTCGGCCGCATCTCA

C

attP$_{skin}$

actgttacgggaaatttatgccccccctttttgtaatgaat**AATGA**gattgtaattacaaaagggggtgcatacacccctctt

attB$_{skin}$

AAGGGACAAAGCTGGCGACGTATGCAGCGAGGTGTATTGAA**AATGA**AATCCTCATGCATTTGCGCGCATTGAAAAAAACAAAAA

attL$_{skin}$ (5'-*skin*)

AAGGGACAAAGCTGGCGACGTATGCAGCGAGGTGTATTGAA**AATGA**gattgtaattacaaaagggggtgcatacacccctctt

attR$_{skin}$ (*skin*-3')

actgttacgggaaatttatgccccccctttttgtaatgaat**AATGA**AATCCTCATGCATTTGCGCGCATTGAAAAAAACAAAAA

D

attP$_{\phi 3T}$

ggtttttcagtttctgcaccatacagtcaataaaaaatgacata**CCTAC**tgtgtttttaaaaataagtatggaaactcctgata

attB$_{\phi 3T}$

AGATCATTGAAGGGCTGAGAGGTCATACCTCAGGCTATGCGGTT**CCTAC**CTTTGTCGTTGACGCACCAGGCGGAGGAGGTAAAA

attL$_{\phi 3T}$ (5'-*kamA*)

AGATCATTGAAGGGCTGAGAGGTCATACCTCAGGCTATGCGGTT**CCTAC**tgtgtttttaaaaataagtatggaaactcctgata

attR$_{\phi 3T}$ (*kamA*-3')

ggtttttcagtttctgcaccatacagtcaataaaaaatgacata**CCTAC**CTTTGTCGTTGACGCACCAGGCGGAGGAGGTAAAA

Figure S1. Nucleotide sequences of attachment sites, related to Figures 1A and 5C. Attachment site sequences of SPβ, (**A**); ICE*Bs1*, (**B**); *skin*, (**C**); and ϕ3T, (**D**). Core sequences that are sites of strand exchanges are indicated by bold characters. Horizontal arrowheads show inverted repeat sequences. Uppercase and lowercase letters indicate host sequences and prophage/ICE sequences, respectively.

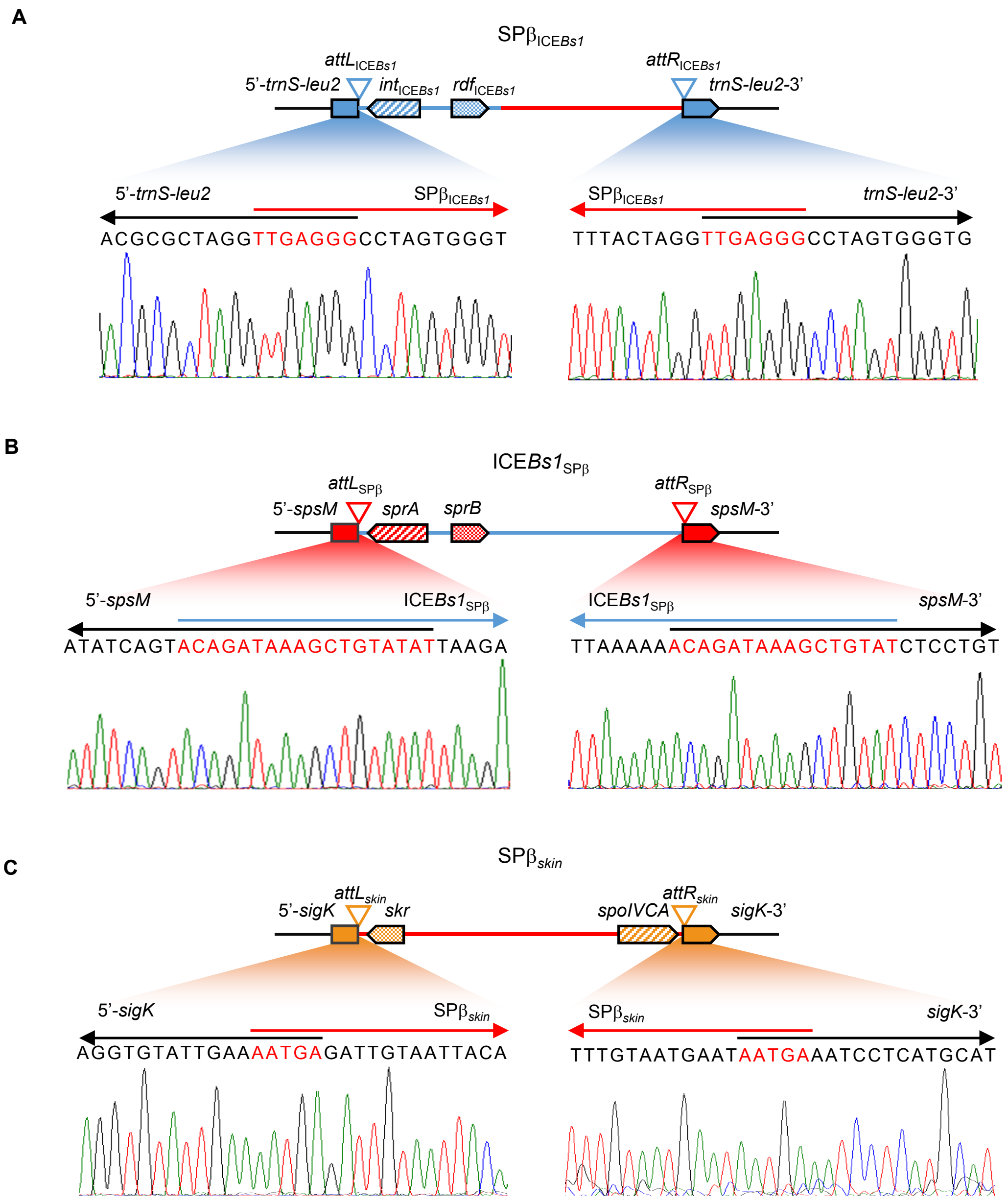

Figure S2. Integration junction sequences of SPβ_{ICEBs1}, ICE*Bs1*$_{SP\beta}$, and SPβ_{skin}, related to Figure 1. *attL* and *attR* sequences of chimeric SPβ_{ICEBs1}, (**A**); ICE*Bs1*$_{SP\beta}$, (**B**); and SPβ_{skin}, (**C**). Above diagrams show the integrated phage or ICE genome into the target genes. Flanking region sequences of each DNA breaking point are shown by the figure below. Core sequences were represented by red character.

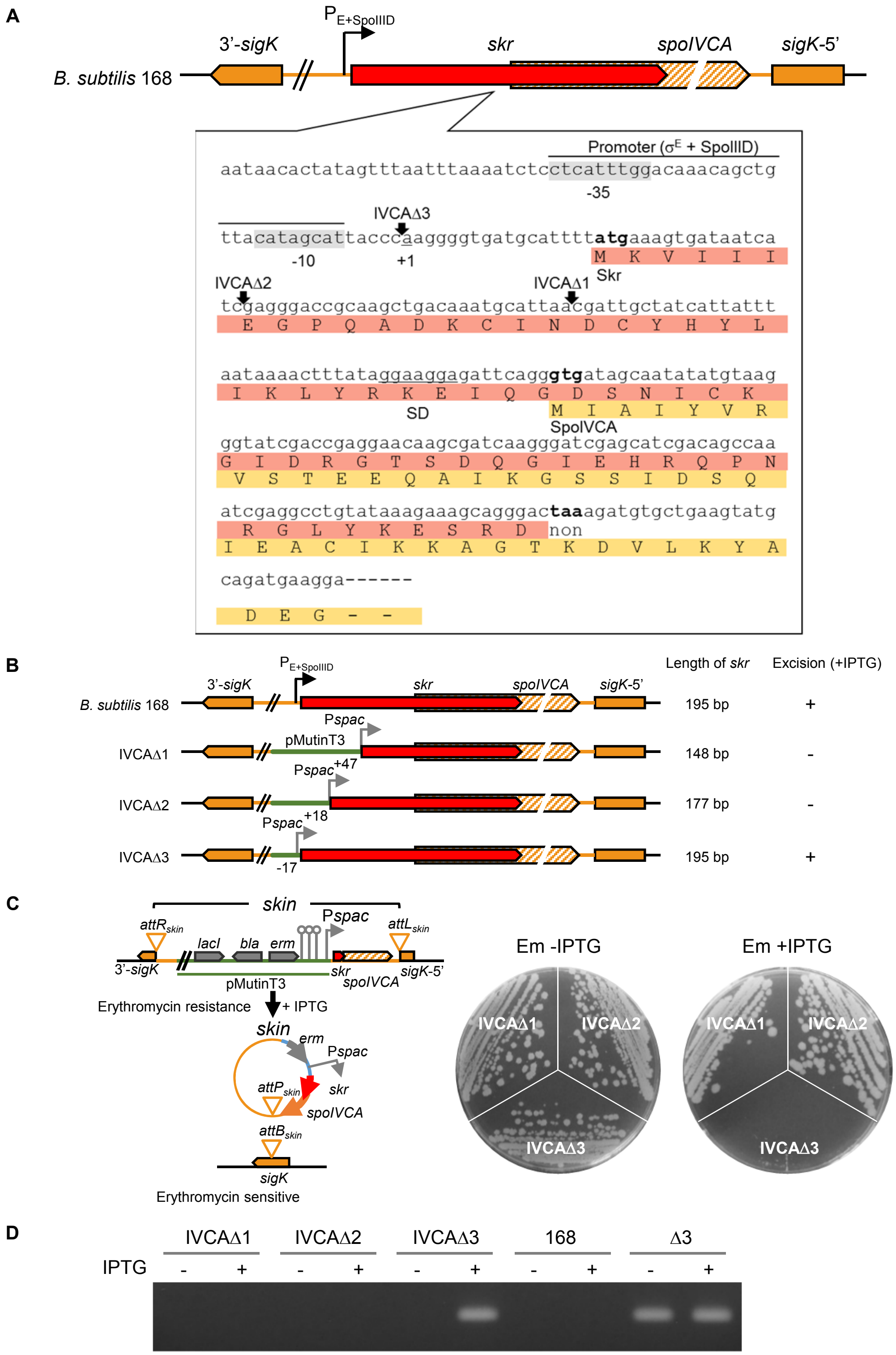

Figure S3. Identification of rdf_{skin} (*skr*) of the *skin* element, related to Figure 3. (**A**), Genomic structure and flanking sequences of *skr*; rdf_{skin} (*skr*) and int_{skin} (*spoIVCA*) genes are arranged in tandem on the chromosome. Vertical arrowheads indicate the insertion positions of the P*spac* promoter. The 3' terminal sequence of *skr* overlapped with 101 bp of the *spoIVCA* gene. Skr and SpoIVCA coding regions are highlighted red and yellow, respectively. (**B**), Schematic of DNA deletion assays for identification of the *skr* gene. An inducible P*spac* promoter was inserted into the positions +47, +18, and −17 from the first nucleotide of the putative *skr* gene and the resulting strains were designated IVCAΔ1, IVCAΔ2, and IVCAΔ3, respectively. (**C**), Excision of *skin* following induction of *skr*. Schematics of *skin* element excision by IPTG are represented in the left panel. The erythromycin resistance gene was excised from the host genome with *skin* after induction of the intact *skr* gene by IPTG treatment. Subsequently, the host strain became sensitive to erythromycin. Growths of IVCAΔ1, IVCAΔ2, and IVCAΔ3 strains on LB agar plates containing erythromycin (Em) with or without IPTG are shown in the right panel. (**D**), Detection of reconstructed $attB_{skin}$. IVCAΔ1, IVCAΔ2, and IVCAΔ3 were grown in LB medium with or without 1 mM IPTG for 16 h. Excision of the *skin* element was confirmed using PCR with the primers P85/P86 and extracted genome templates.

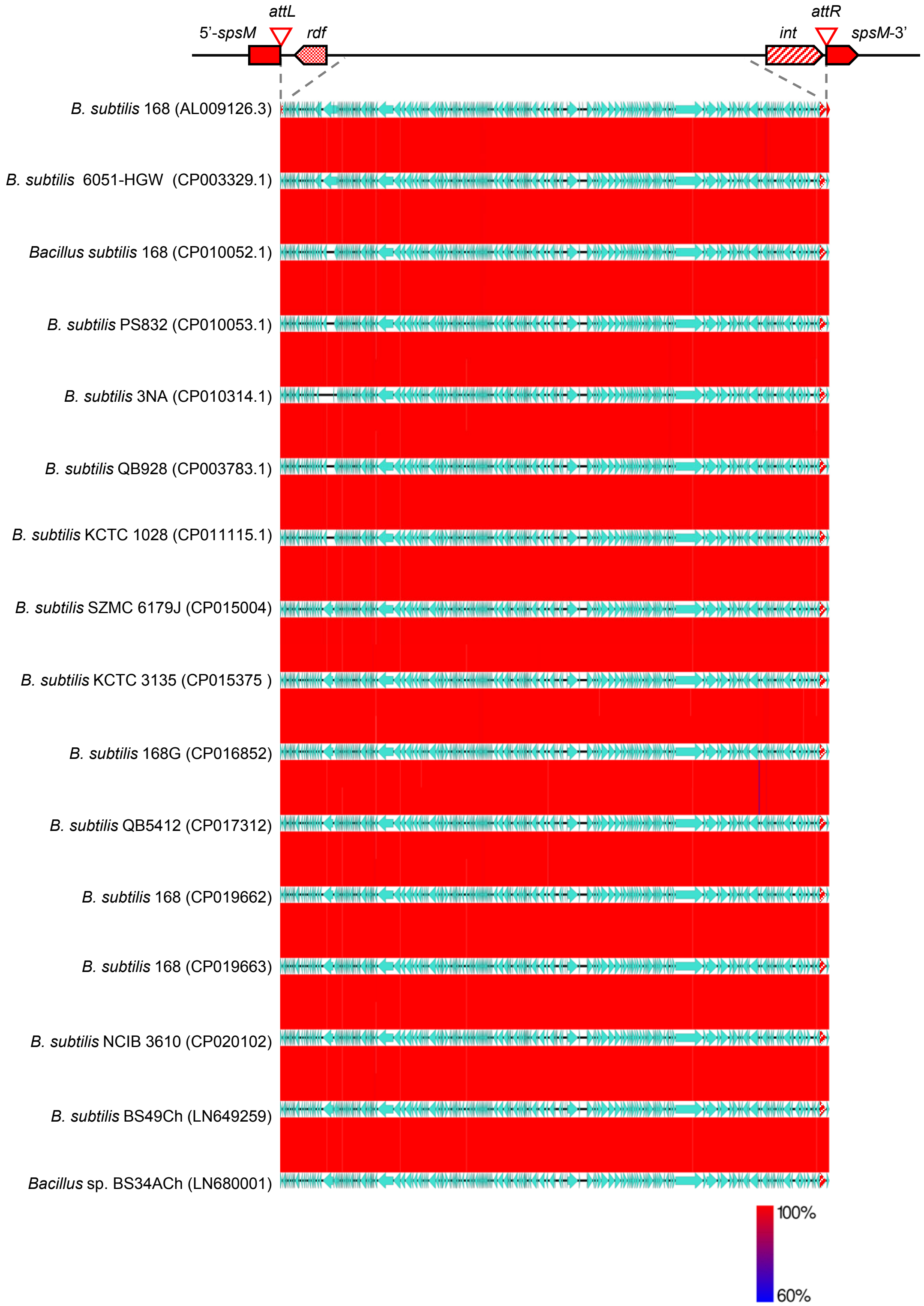

Figure S4. Synteny plots of SPβ related phages using Easyfig tBLASTx, related to Figure 4. Genome comparison of sixteen SPβ related phage genomes possessing an SSR unit homologous to that of SPβ (*B. subtilis* 168) and residing in the *spsM* gene. Host names and accession numbers are indicated on the left column. Blue-Red lines indicate region with 60-100 % identity.

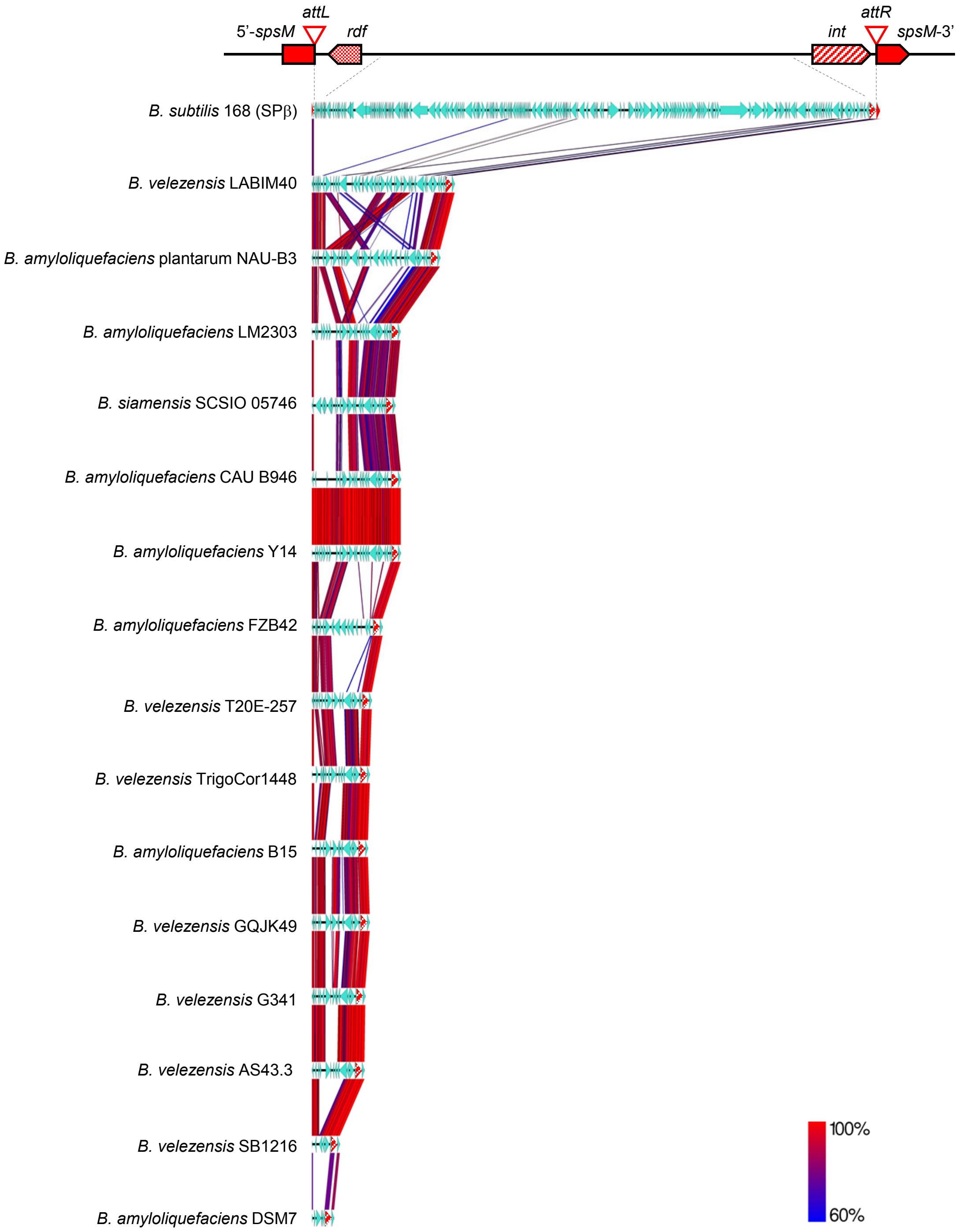

Figure S5. Synteny plots of degenerate SPβ phages from Easyfig tBLASTx, related to Figure 4. Genome comparisons of fifteen degenerate SPβ phage genomes carrying SSR units homologous to that of SPβ (*B. subtilis* 168) and residing in the *spsM* gene; host names are indicated on the left. Blue–red lines indicate regions with 60%–100% identity.

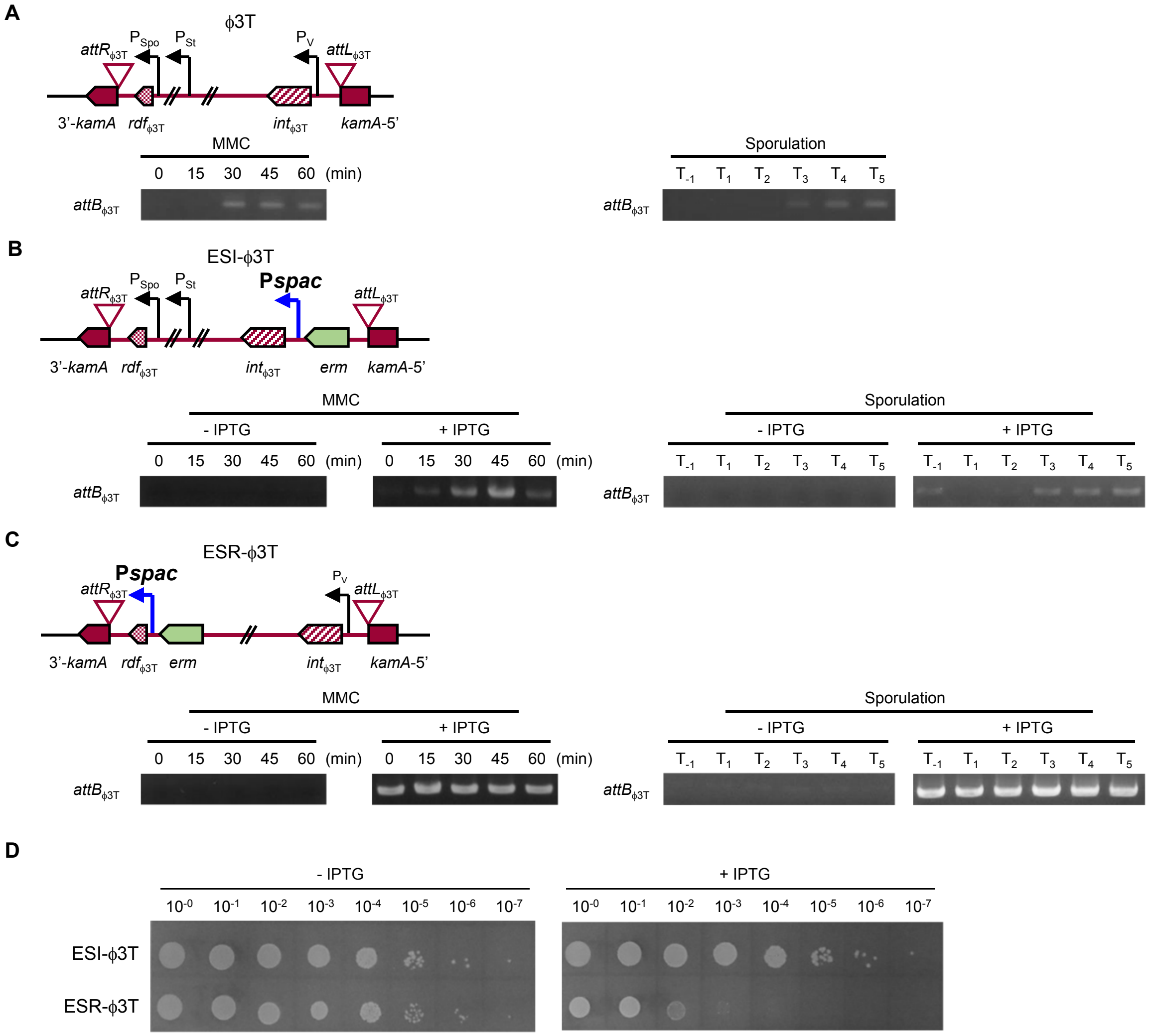

Figure S6. ϕ3T excision upon induction of $int_{\phi 3T}$ or $rdf_{\phi 3T}$, related to Figure 5. (**A**) Detection of ϕ3T excision. $attB_{\phi 3T}$ (229 -bp) was amplified using PCR with the primers P79/P80. Detection of ESI-ϕ3T excision, (**B**) and ESR-ϕ3T excision, (**C**), upon MMC treatment and during sporulation with or without IPTG. $attB_{\phi 3T}$ (1447-bp) was PCR amplified using the primers P65/P81 for B and C. Positions and directions of native and P*spac* promoters are represented by black and blue arrowheads, respectively. (**D**), Detection of ϕ3T genome excision by antibiotic selection on plates. Ten-fold serial dilutions of cultures were spotted onto LB agar plates containing erythromycin with or without IPTG. Horizontal arrowheads represent positions and directions of transcriptional promoters; P_V, vegetative promoter; P_{spo}, sporulation-specific promoter; P_{St}, stress inducible promoter; P*spac*, IPTG-inducible promoter.

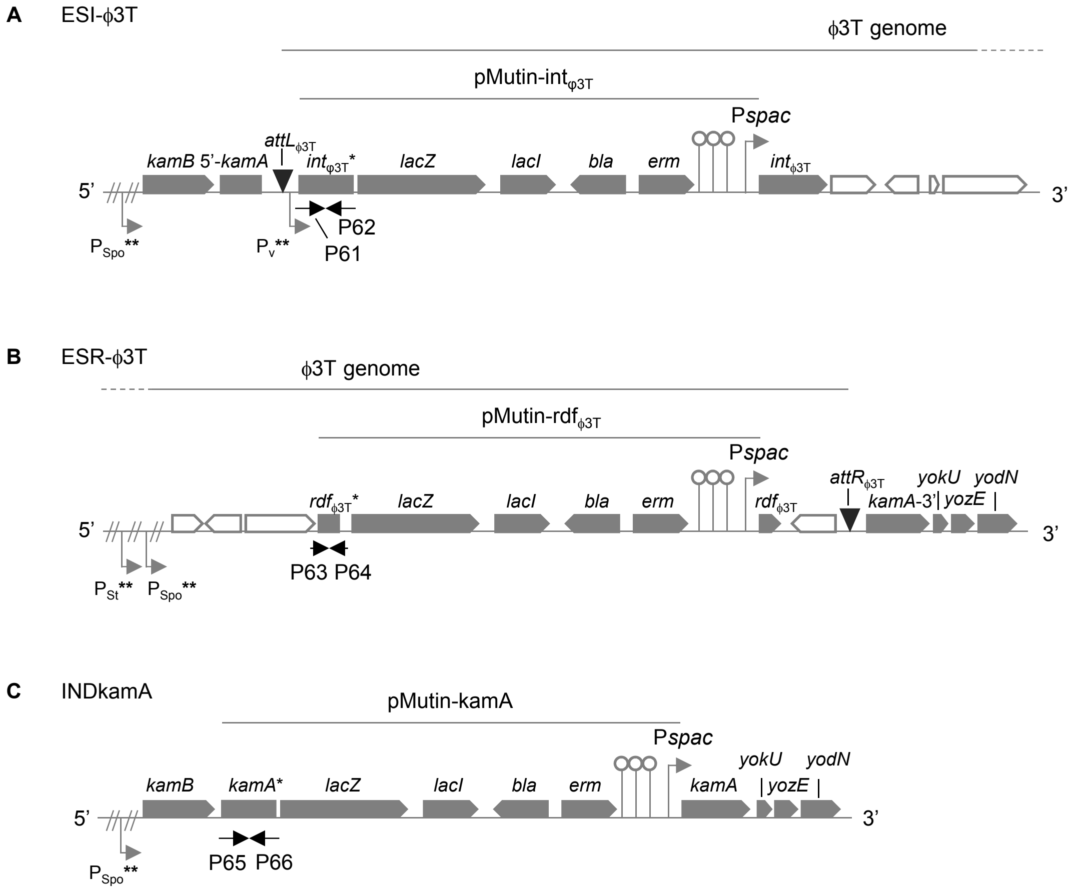

Figure S7. Construction of ESI-ϕ3T, ESR-ϕ3T, and INDkamA, related to Figure 5. Schematics of construction of ESI-ϕ3T, (**A**); ESR-ϕ3T, (**B**); and INDkamA, (**C**), strains. Gene names are indicated. The horizontal gray arrowheads and horizontal black arrowheads indicate the position of P*spac* promoter and of primers used for construction of pMutin-int$_{\phi 3T}$, pMutin-rdf$_{\phi 3T}$, and pMutin-kamA, respectively. The horizontal gray arrowheads indicate putative position and direction of native transcriptional promoters. P_V, σ^A-dependent promoter; P_{Spo}, sporulation specific promoter; P_{St}, stress inducible promoter. Transcriptional terminators are presented by open circles. Single and double asterisks indicate disrupted genes and putative promoters, respectively. Open boxes are represented ϕ3T phage genes excluding $int_{\phi 3T}$ and $rdf_{\phi 3T}$.

Supplemental Tables

Table S1. Relevant characteristics of chimeric phages and ICEs, related to Figure 1.

Mobile element	Genome size kb	Marker	*attL*	*rdf*	*int*	*attR*
SPβ	134		$attL_{\mathrm{SP\beta}}$	*sprB*	*sprA*	$attR_{\mathrm{SP\beta}}$
ICE*Bs1*	20		$attL_{\mathrm{ICE}Bs1}$	$xis_{\mathrm{ICE}Bs1}$	$int_{\mathrm{ICE}Bs1}$	$attR_{\mathrm{ICE}Bs1}$
skin	48		$attL_{skin}$	*skr*	*spoIVCA*	$attR_{skin}$
$\mathrm{SP\beta}_{kan}$	136	*kan*	$attL_{\mathrm{SP\beta}}$	*sprB*	*sprA*	$attR_{\mathrm{SP\beta}}$
$\mathrm{SP\beta}_{\mathrm{ICE}Bs1}$	137	*erm*	$attL_{\mathrm{ICE}Bs1}$	$xis_{\mathrm{ICE}Bs1}$	$int_{\mathrm{ICE}Bs1}$	$attR_{\mathrm{ICE}Bs1}$
$\mathrm{ICE}Bs1_{cat}$	22	*cat*	$attL_{\mathrm{ICE}Bs1}$	$xis_{\mathrm{ICE}Bs1}$	$int_{\mathrm{ICE}Bs1}$	$attR_{\mathrm{ICE}Bs1}$
$\mathrm{ICE}Bs1_{\mathrm{SP\beta}}$	22	*cat*	$attL_{\mathrm{SP\beta}}$	*sprB*	*sprA*	$attR_{\mathrm{SP\beta}}$
$\mathrm{SP\beta}_{skin}$	136	*spc*	$attL_{skin}$	*skr*	*spoIVCA*	$attR_{skin}$

Table S2. Information of integrase and rdf protein sequences, related to Figures 1, 4, and 5.

Phage/ICE	Protein	Accession ID
SPβ	SprA	CAB14084.1
SPβ	SprB	CAB13873.1
ϕ3T	$Int_{\phi 3T}$	APD21144.1
ϕ3T	$RDF_{\phi 3T}$	APD21343.1.
ϕ12-5	$Int_{\phi 12\text{-}5}$	AMR46776.1
ϕ12-5	$RDF_{\phi 12\text{-}5}$*	CP014858<br>From 2027172 to 2027318
ICE*Bs1*	Int	CAB12287.1
ICE*Bs1*	Xis	CAB12290.1
skin	SpoIVCA	CAB14518.2
skin	Skr	AL009126<br>From 2654774 to 2654968

* Predicted

Table S3. Mating frequency of ICE*Bs1*$_{cat}$ and chimeric ICE*Bs1*$_{SPβ}$, related to Figure 1.

ICEs	Conjugation freq.[a]	Integrated at *attB* sites (%)[b]
ICE*Bs1*$_{cat}$	3.3 (± 0.7) × 10^{-2}	100
ICE*Bs1*$_{SPβ}$	3.3 (± 1.7) × 10^{-2}	100

[a] The data shown are the average of three independent experiments ± SD.

[b] 10 transconjugants were investigated.

Table S5. Strains constructed in this study, related to Figures 1 and 5 and Transparent Methods.

Strains	Genotype and/or Characteristics	Source or Reference
HSS001	*trpC2* SPβ$_{ICEBs1}$ Δ*sprB*::*erm*	This study
HSS002	*trpC2* SPβ cured strain, *yddM*::*cat*	This study
HSS003	*trpC2* SPβ cured strain, ICE*Bs1*$_{SPβ}$ *yddM*::*cat*	This study
HSS004	*trpC2* SPβ$_{skin}$ *yokB*::*spc*	This study
HSS005	*trpC2* SPβ$_{kan}$ *yokB*::*kan*	This study
ESI-ϕ3T	*trpC2* ϕ3T Δ*int*::pMutinT3-int$_{ϕ3T}$ *erm*	This study
ESR-ϕ3T	*trpC2* ϕ3T Δ*rdf*::pMutinT3-rdf$_{ϕ3T}$ *erm*	This study
INDkamA	*trpC2* Δ*kamA*::pMutinT3-kamA *erm*	This study
Δ2	*trpC2* SPβ and ICE*Bs1* cured strain	This study
Δ3	*trpC2* SPβ, ICE*Bs1*, and *skin* element cured strain	This study
Δ2CK	*trpC2* ICE*Bs1* and SPβ cured strain, Δ*comK*::*kan*	This study
IVCAΔ1	*trpC2 skr*::pIVCAΔ1 *erm*	This study
IVCAΔ2	*trpC2 skr*::pIVCAΔ2 *erm*	This study
IVCAΔ3	*trpC2 skr*::pIVCAΔ3 *erm*	This study

Transparent methods

Growth media

Standard genetic manipulations of *B. subtilis* were performed as described previously **(Harwood and Cutting, 1990)**. Cells were grown at 37°C with shaking in Luria-Bertani medium (LB) **(Sambrook and Russell, 2001)**, Difco sporulation medium (DSM) **(Harwood and Cutting, 1990)**, and defined minimal medium **(Auchtung et al., 2005)** supplemented with 50 μg/ml tryptophan. When required, antibiotics were added at the following concentrations: chloramphenicol, 5 μg/ml; erythromycin, 0.5 μg/ml; kanamycin, 5 μg/ml; spectinomycin, 100 μg/ml; ampicillin, 100 μg/ml.

Strain construction

All strains were derived from *B. subtilis* 168. The primers and the bacterial strains used in this study are listed in Tables S4 and S5, respectively. Q5 High-Fidelity DNA polymerase (NEB, U.S.A) was used to construct donor DNA fragments and plasmids.

To construct chimeric SP$\beta_{\mathrm{ICE}\mathit{Bs1}}$ phages, primer pairs P1/P2, P3/P4, and P5/P6 were used to amplify regions from *cgeB* to 5'-*spsM* genes, from $attL_{\mathrm{ICE}\mathit{Bs1}}$ to *ydcO* genes, and from $attL_{\mathrm{SP}\beta}$ to *yotJ* genes in the SPRBd **(Abe et al., 2014)** genome, respectively. The *B. subtilis* 168 genome was used as a template for P1/P2 and P3/P4 amplifications. The obtained DNA fragments were simultaneously used as templates for PCRs with the primers P1/P6. The four primer pairs P7/P8, P9/P10, P11/P12, and P13/P14 were used to amplify the region from *yokC* to *yokB*, $attR_{\mathrm{ICE}\mathit{Bs1}}$, a spectinomycin resistant gene of pUCS191 **(Hosoya et al., 2002)**, and a region from *spsM*-3' to *msrA*, respectively. The *B. subtilis* 168 genome was used as a template for PCR amplification with primers P7/P8, P9/P10, and P13/P14. The obtained DNA fragments were simultaneously used as templates for PCR with the primer pair P7/P14. The resulting products from P1/P6 and P7/P14 primer pairs were used to transform *B. subtilis* 168 and erythromycin- and spectinomycin resistant transformants were selected, resulting in strain HSS001.

To select conjugated ICE*Bs1*, a chloramphenicol resistance gene was introduced within the *yddM* gene and the $attR_{\mathrm{ICE}\mathit{Bs1}}$ region in ICE*Bs1*. The primer pairs P15/P16, P17/P18, and P19/P20 were used to amplify *rapI* to *yddM*, a chloramphenicol resistant gene (*cat*) from pMF20 **(Murakami et al., 2002)**, and $attR_{\mathrm{ICE}\mathit{Bs1}}$ to *lrpA*, respectively. The *B. subtilis* 168 genome was used as template for PCR with primer pairs P15/P16 and P19/P20. PCR products were then simultaneously used for amplification with the primers P15/P20. The

resulting products were used to transform SPβless. Chloramphenicol resistant cells were selected and the resulting strain was designated HSS002.

To construct chimeric ICE*Bs1*$_{\text{SP}\beta}$, the primer pairs P21/22, P23/P24, P25/P26, P27/P28, P29/P30, P31/P32, and P33/P34 were used to amplify the region from *ydcI* to *trnS-leu1*, the erythromycin resistant gene of pUCE191 **(Abe et al., 2014)**, spacer sequences between *trnS-leu1* and *attL*$_{\text{ICE}Bs1}$, from *sprA* to *attR*$_{\text{SP}\beta}$, from *immA* to *immR*, the *sprB* gene, and from *ydcL* to *ydcQ*, respectively. The *B. subtilis* 168 genome was used as template for P21/P22, P25/P26, P27/P28, P29/P30, P31/P32, and P33/P34 amplifications. The resulting DNA fragments were simultaneously used in PCR amplifications with primers P21/P34. Primer pairs P35/P36, P37/P38, and P39/P40 were used to amplify the region from *rapI* to the *cat* gene in the HSS002 genome, *attL*$_{\text{SP}\beta}$, and *yddN* and *lrpA* genes, respectively. The *B. subtilis* 168 genome was used as template for PCR with P37/P38 and P39/P40 primers. The resulting DNA fragments were simultaneously used in PCR with the primer pair P35/P40. The resulting DNA fragments were used to transform SPβless cells. The erythromycin and chloramphenicol resistant strain was selected and designated HSS003.

To eliminate the possibility of ICE*Bs1* integration in *B. subtilis* cells, the *comK* gene was disrupted. To this end, we used the genome of the 8G32 (Δ*comK*::*kan*) **(Ogura and Tanaka, 1997)** strain to transform the ICE*Bs1*-less SPβ-less strain (Δ2), which was designated Δ2CK.

To construct chimeric SPβ$_{skin}$, the primer pairs P41/P42, P43/P44, P45/P49, and P50/P6 were used to amplify the region from *phy* to 5'-*spsM*, the kanamycin resistant gene (*kan*) of pJM114 **(Perego, 1993)**, the *skr* gene of the *skin* element, and the region from *yotM* to *yotJ*, respectively. The *B. subtilis* 168 genome was used as template for PCR with P41/P42, P45/P49, and P50/P6 primer pairs. The fragment from PCR with the P45/P49 primer pair was used as a template for step-by-step PCR using primer pairs P46/P49, P47/P49, and P48/P49 in order to attach an *attL*$_{skin}$ sequence to the 5' end of the fragment. These fragments were simultaneously used as template in PCR with the primers P41/P6. The primer pairs P51/P52, P11/P12, P53/P54, P55/P56, and P57/P14 were used to amplify the region from *yokC* to *yokB*, the spectinomycin resistant gene of pUCS191, the promotor region of *sprA*, from *spoIVCA* to *attR*$_{skin}$, and from *spsM*-3' to *msrA*, respectively. The *B. subtilis* 168 genome was used as template for PCR with the primer pairs P51/P52, P53/P54, P55/P56, and P57/P14. These fragments were simultaneously amplified using primer pair P51/P14. The fragments from P41/P6 and P51/P14 primer pairs were used to transform the *B. subtilis* 168 strain, resulting in the strain designated HSS004.

SPβ$_{kan}$ was constructed as follows: primer pairs P7/P58, P43/P44, and P59/P60 were used to amplify the region from *yokC* to *yokB*, the *kan* gene of pJM114, and the region from *sprA* to *msrA*, respectively. The *B.*

subtilis 168 genome was used as template for PCR with the primer pairs P7/P58 and P59/P60. The resulting fragments were simultaneously used as templates in PCR with the primer pair P7/P60. The resulting DNA fragment was used to transform the *B. subtilis* 168 strain to produce the strain HSS005.

The ϕ3T lysogen was constructed by infecting SPβless cells with ϕ3T phages. Integration of ϕ3T was confirmed by PCR amplification using the primers P65/P82 for $attL_{\phi3T}$ and P81/P87 for $attR_{\phi3T}$, followed by DNA sequence analyses. To construct $int_{\phi3T}$ or $rdf_{\phi3T}$-inducible strains, $int_{\phi3T}$ (−31 to +958 relative to the first nucleotide of the start codon) and $rdf_{\phi3T}$ (−24 to +91) were amplified from the chromosome of ϕ3T lysogens using the primers P61/P62 and P63/P64, respectively. The obtained DNA fragments were digested using *Bam*HI and *Hin*dIII and were inserted into the *Bam*HI-*Hin*dIII site of pMutinT3 **(Vagner et al., 1998)**. The resulting pMutinT3-$int_{\phi3T}$ and pMutinT3-$rdf_{\phi3T}$ constructs were used to transform the ϕ3T lysogen and the corresponding $int_{\phi3T}$ or $rdf_{\phi3T}$-inducible strains were designed ESI-ϕ3T and ESR-ϕ3T, respectively (Figures S7A and B). To construct a *kamA*-inducible strain, the *kamA* gene was PCR amplified using the primer pair P65/P66 and the amplicon was digested by *Bam*HI and *Hin*dIII and inserted into *Bam*HI-*Hin*dIII site of the plasmid pMutinT3. Subsequently, the pMutin-kamA plasmid was used to transform the SPβ-less strain to produce the INDkamA strain (Figure S7C).

SPβ and ICE*Bs1* cured strains (Δ2) were constructed by amplifying xis_{ICEBs1} to *ydzL* genes using the primers P67/P68. Amplicons were then digested using *Hin*dIII and *Bam*HI and were ligated into the *Hin*dIII/*Bam*HI sites of linearized pMutinT3 plasmid. The resulting plasmid construct was used to transform SPless. Transformants were selected according to erythromycin resistance on LB agar plates containing 1-mM IPTG for 16 h. Erythromycin resistance was confirmed by curing ICE*Bs1* by PCR amplification using the primer pair P75/P76. The resultant strain was designated Δ2 (*trpC2* SPβless ICE*Bs1*less).

Strains for identification of rdf_{skin} were constructed as follows: The primer pairs P69/P70, P69/ P71, and P69/P72 were used to amplify upstream of *spoIVCA* from −47 to +282, −76 to +282, and −111 to +282 relative to the first nucleotide of *spoIVCA*, respectively. The *B. subtilis* 168 genome was used as a template. Amplified DNA fragments were digested with *Hin*dIII and *Bam*HI and were ligated into the *Hin*dIII/*Bam*HI sites of linearized pMutinT3 plasmid. The resulting plasmids, pIVCAΔ1, pIVCAΔ2, and pIVCAΔ3 plasmids were used to transform *B. subtilis* 168. Transformants were selected according to erythromycin resistance and were designated IVCAΔ1, IVCAΔ2, and IVCAΔ3 respectively.

SPβ-less ICE*Bs1*-less and *skin*-less (Δ3) strains were constructed as follows: initially the Δ2 strain was transformed with the plasmid pIVCAΔ3 and transformants were then grown on LB agar plates containing 1-

mM IPTG for 16 h. The resulting erythromycin resistant strain was confirmed as SPβless, ICE*Bs1*-less, and *skin*-less strain using PCR and was designated Δ3 (*trpC2* SPβless ICE*Bs1*-less *skin*-less).

These constructed strains were confirmed by PCR and DNA sequence analysis.

Phage preparation

SPβ, $SP\beta_{kan}$, $SP\beta_{ICEBs1}$, $SP\beta_{skin}$, and ϕ3T phage lysates were prepared from 168, HSS005, HSS001, HSS004, and BGSC 1L1 (CU1065 ϕ3T) strains, respectively. Phage lysogens were precultured overnight in LB medium at 30°C with shaking. Overnight cultures were then diluted 100-fold in LB medium and were grown to the early log phase (OD_{600} ~ 0.2). Cultures were then incubated at 37°C in the presence of MMC (0.5 µg/ml) and when the optical density was decreased to around OD_{600} ~ 0.1, cells were removed by centrifugation at 7,000 × *g* at 4°C and lysates were filtered through 0.45-µm membrane filters. Phage lysates were then stored at 4°C and were spotted onto lawns of Δ3 strains to evaluate their abilities to form phage plaques.

Measurements of integration frequencies

The Δ3 strain was grown to early log phase (OD_{600} ~ 0.2) in LB medium and was infected with the obtained phages at a multiplicity of infection (MOI) of 0.1. Cells were then incubated for 1 h at room temperature without shaking, and were then plated onto LB plates containing antibiotics. Integration frequencies were calculated as described by Tal et al. **(2014)**. Insertion of the phages into *attB* sites was verified using colony PCR. In these analyses, colonies were picked using sterilized toothpicks and were transferred to PCR tubes. PCR tubes were then irradiated in a microwave oven for 1.5 min and PCR reaction mixture was added to the PCR tubes.

Mating experiments

Mating experiments were performed using previously published methods **(Auchtung et al., 2005)** with some modifications. Briefly, donor cells were grown in defined minimal medium and treated with MMC. Transconjugants were selected according to the presence of kanamycin and chloramphenicol resistance genes in $ICEBs1_{cat}$ and $ICEBs1_{SP\beta}$. Mating frequencies were calculated by dividing numbers of transconjugants by numbers of recipient cells. Transfer frequencies are reported as means ± standard errors of the mean from at least three independent biological replicates. Insertion of phages into *attB* sites was verified using colony PCR.

β-galactosidase assays

Insertion of pMutinT3 plasmid into target genes inactivates the gene and allows analysis of its transcriptional profile by measuring β-galactosidase activity. To measure transcriptional activity of $int_{\phi 3T}$, $rdf_{\phi 3T}$, and *kamA*, the strains ESI-ϕ3T, ESR-ϕ3T, and INDkamA strains, respectively, were precultured in LB medium at 30°C for 16 h. Cells were then inoculated into Difco sporulation medium (DSM) at OD_{600} ~ 0.04 and were incubated at 37°C for indicated times. β-galactosidase activity was determined using the method described by Miller **(Miller, 1972)**.

Excision assay

To evaluate phage and ICE excision from host genomes, *attB*, but not *attP*, was used as a target DNA region for PCR amplification, because under these conditions, phage and ICE genomes were spontaneously excised at low frequencies and were amplified into multiple copies of DNA. PCR amplification was performed using Prime taq (GenetBio, Korea) with 100-ng aliquots of extracted genomic DNA. Primer sequences are listed in Table S4. PCR cycle numbers were adjusted to avoid reaching plateaus as follows: P73/P74 for $attB_{SP\beta}$, $SP\beta_{kan}$ and SPβ, 25 cycles; $ICEBs1_{SP\beta}$, 27 cycles; P75/P76 for $attB_{ICEBs1}$, $SP\beta_{ICEBs1}$, 27 cycles; $ICEBs1_{cat}$, 25 cycles; P77/P78 for $attB_{skin}$, *skin* and $SP\beta_{skin}$, 25 cycles.

Quantitative PCR assays

Quantitative PCR assays were performed using previously published methods **(Abe et al., 2017**) with some modifications. Briefly, the qPCR reactions were performed using the KOD SYBR qPCR Mix (TOYOBO, Japan) with 50-ng aliquots of genomic DNA. To prepare a DNA standard for absolute quantification of *attB*, the $attB_{SP\beta}$, $attB_{ICEBs1}$, $attB_{skin}$, and $attB_{\phi 3T}$ were amplified by PCR from the chromosomal DNA of Δ3 strain, using P73/P74, P75/P76, P77/P78, and P79/P80, respectively. The quantitative PCR assay was conducted at 98°C for 2 min and then 40 cycles of 98°C for 10 sec, 60°C for 10 sec, and 68°C for 35 sec. The reaction specificity was verified using a melt curve analysis. As an internal control for the quantification, the copy number of *yodT*, which is a single-copy gene in the *B. subtilis* genome with no involvement in the phage excision, was quantified by the same method as described above, using the P83/P84 primers. The phage and ICE excision frequency were calculated as a ratio of the copy number of the *attB* site to that of *yodT*.

Synteny plots of SMGC–Easyfig

Synteny plots were generated using Easyfig version 2.2.2 **(Sullivan et al., 2011)**. All GenBank files describing the clusters to be compared were obtained from the nucleotide data base of the National Center for Biotechnology Information (NCBI). The tBLASTx option was used with an e− ≤ 0.001 and an alignment identity of ≥ 60.

Supplemental references

Harwood, C.R., and Cutting, S.S. (1990). Molecular Biological Methods for *Bacillus*. (John Wiley & Sons Ltd, Chichester).

Hosoya,S., Asai,K., Ogasawara,N., Takeuchi,M. and Sato,T. (2002) Mutation in *yaaT* leads to significant I nhibition of phosphorelay during sporulation in *Bacillus subtilis*. J. Bacteriol. *184*, 5545-5553.

Miller, J.M. (1972). Experiments in molecular genetics. (New York: Cold Spring Harbor Laboratory Press), pp. 352–355.

Murakami,T., Haga,K., Takeuchi,M. and Sato,T. (2002) Analysis of the *Bacillus subtilis spoIIIJ* gene and its paralogue gene, *yqjG*. J. Bacteriol. *184*, 1998–2004.

Ogura,M. and Tanaka,T. (1997) *Bacillus subtilis* ComK negatively regulates *degR* gene expression. *Mol. Gen. Genet*. *254*, 157-165.

Perego,M. (1993) Integrational Vectors for Genetic Manipulation in *Bacillus subtilis*: *Bacillus subtilis* and other gram-positive bacteria: biochemistry, physiology, and molecular genetics. Washington, D.C.: American Society for Microbiology.

Sambrook, J. and Russell, D.W. (2001). Molecular Cloning: A Laboratory Manual. (New York: Cold Spring Harbor Laboratory Press).

Sullivan, M.J., Petty, N.K., and Beatson, S.A. (2011). Easyfig: a genome comparison visualizer. Bioinformatics *27*, 1009-1010.

Tal, A., Arbel-Goren, R., Costantino, N., Court, D.L., and Stavansa, J. (2014). Location of the unique integration site on an Escherichia coli chromosome by bacteriophage lambda DNA in vivo. Proc. Natl. Acad. Sci. USA *111*, 7308-7312.

Vagner,V., Dervyn,E. and Ehrlich,S.D. (1998) A vector for systematic gene inactivation in *Bacillus subtilis*. Microbiology *144*, 3097–3104.
